# Fire behaviors along timber linings affixed to tunnel walls in mines

**DOI:** 10.1371/journal.pone.0260655

**Published:** 2021-12-02

**Authors:** Ke Gao, Zimeng Liu, Changfa Tao, Zhiqiang Tang, Yisimayili Aiyiti, Lianzeng Shi

**Affiliations:** 1 College of Safety Science and Engineering, Liaoning Technical University, Huludao, Liaoning, China; 2 Centre for Built Infrastructure Research, School of Civil and Environmental Engineering, University of Technology, Sydney, NSW, Australia; 3 Key Laboratory of Mine Thermo-Motive Disaster and Prevention, Ministry of Education, Huludao, Liaoning, China; 4 College of Automotive and Traffic Engineering, Hefei University of Technology, Hefei, China; University of New South Wales, AUSTRALIA

## Abstract

Timber linings are applied as primary supports in the tunnel fault and fracture zones of mines. These linings are essential to prevent broken rock from falling during the occurrence of exogenous fires. In this study, experiments and numerical simulations were carried out using a fire dynamics simulator to investigate the flame-spread rate, flame characteristics, smoke movement, and spread process of timber-lining fires under different wind speeds of 0, 0.25, 0.5, and 0.75 m/s. It was found that cross-section flame spreading follows the three-stage sidewall-ceiling-sidewall pattern. Moreover, the average flame-spread rate increases along the vertical flame-spreading direction and decreases when the flame reaches the timber-lining corners. Moreover, the flame lengths underneath the timber-lining ceiling in the x-direction are longer than those in the y-direction. As the wind speed increases, the normalized flame lengths *R*(*f*) in the two directions decrease, and the maximum temperature underneath the ceiling decreases. In addition, the maximum temperature in the three tunnel sections of interest is first recorded in the tunnel cross-section in the initial fire stage. Higher wind speeds correspond to farther distances of the maximum-temperature points of the three timber-lining sections from the fire source.

## 1. Introduction

The five major disasters that can occur in mines are fire, gas disasters, flood, dust, and roof collapse [1–3]. As regards fire occurrence, several combustible materials such as belts, cables, and timber are present in tunnels, which can cause exogenous mine fires [4, 5]. It is noteworthy that the exogenous-fire-spread process and mechanism are complex owing to the enclosed space in mines [6]. Thus, many combustible materials have been replaced with alternatives to prevent exogenous fires. Timber supports have been replaced by U-shaped steel or bolt-shotcrete supports; however, timber is still widely used as lining material to prevent rock collapse in the tunnel fault and fracture zones. These linings act as fire hazards in tunnels, and they may easily cause timber fires, leading to substantial economic losses and casualties. In this regard, several cases of mine fires resulting from timber linings have been reported. There were two similar timber-lining fires in mine tunnels in China: on December 17, 2015, an electric welder accidentally ignited the timber linings, resulting in 17 deaths, 17 injuries, and a loss of $3 million in a mine owned by Liaoning Lianshan Molybdenum Industry Co., Ltd. On August 16, 2016, a similar timber-lining fire occurred in a mine owned by Gansu Jiu Steel Group Hongxing Iron & Steel Co., Ltd., resulting in 12 deaths, 17 injuries, and losses of $2.8 million. Consequently, it is urgently required to study the characteristics of timber-lining fires in underground mines.

In this context, previous works have mainly focused on smoke propagation [7–13], temperature distribution [14–17], and other influential factors [18] at play in underground mines or tunnels used for vehicular traffic. Furthermore, solutions such as the use of brattice curtains, conveyor belts [19], electrical insulating materials [20, 21], and mining vehicles [22, 23] have been investigated. However, little research has been conducted on the fire behavior related to timber linings in tunnels. Meanwhile, several studies have focused on timber fires in closed rooms. Nishino and Kagiya [24] studied the pre-flashover fire behavior in a room with combustible timber linings to predict the gas temperature using the wall-and-ceiling flame-spread model. Hadden [25] conducted a series of compartment fire experiments to evaluate the impact of combustible cross-laminated timber linings on the resulting fires. Tao [26, 27] studied the effects of fire plumes near the wall on the characteristics of air entrainment, flame height, and virtual origins. In summary, the flammability of timber linings has necessitated research on timber-lining fires.

In this study, the general fire behaviors of timber-lining fires are examined and discussed based on experiments and numerical simulations. This study analyzes the smoke movement, temperature distribution, and flame characteristics of timber-lining fires under different wind speeds. The study findings can provide important insights into the safety design of mining tunnels.

## 2. Experimental setup and discussion

### 2.1 Experimental apparatus and procedure

**Fig 1:**
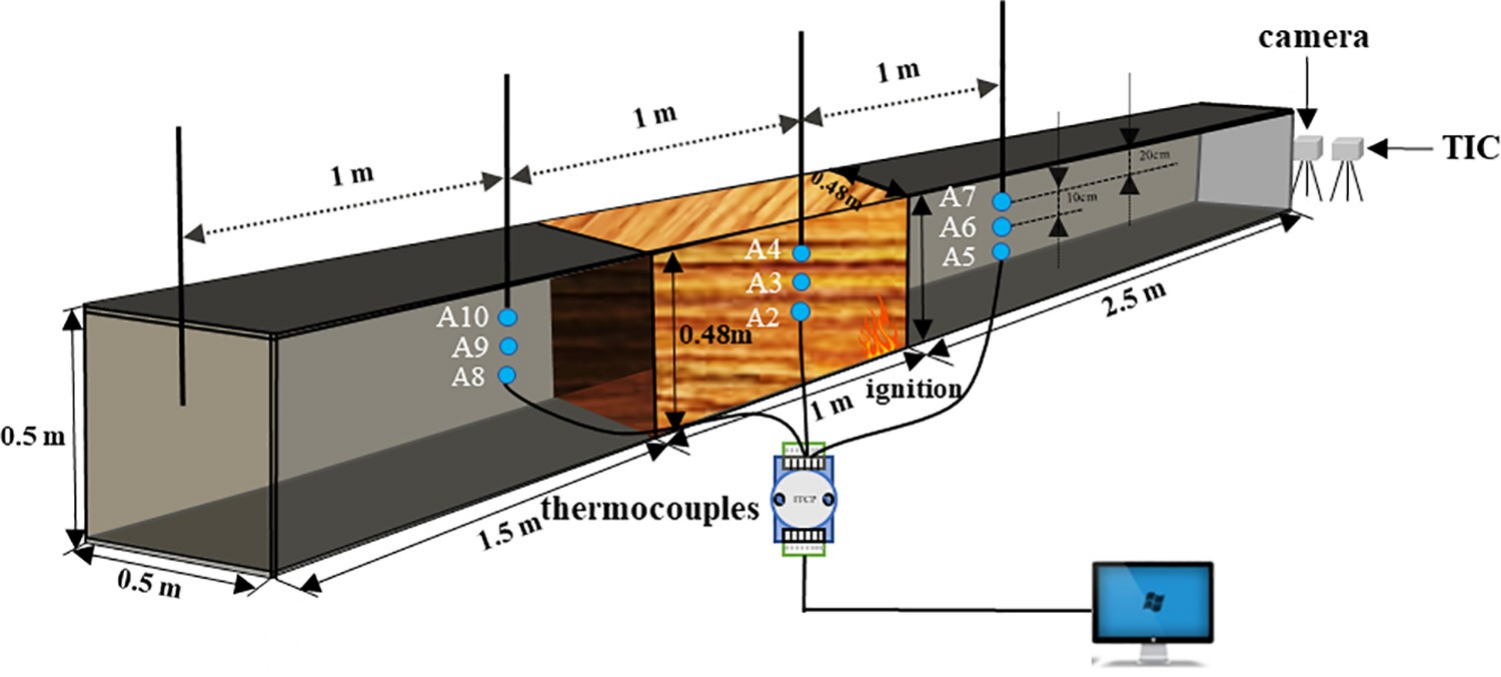
Schematic of tunnel model.

**Fig 2:**
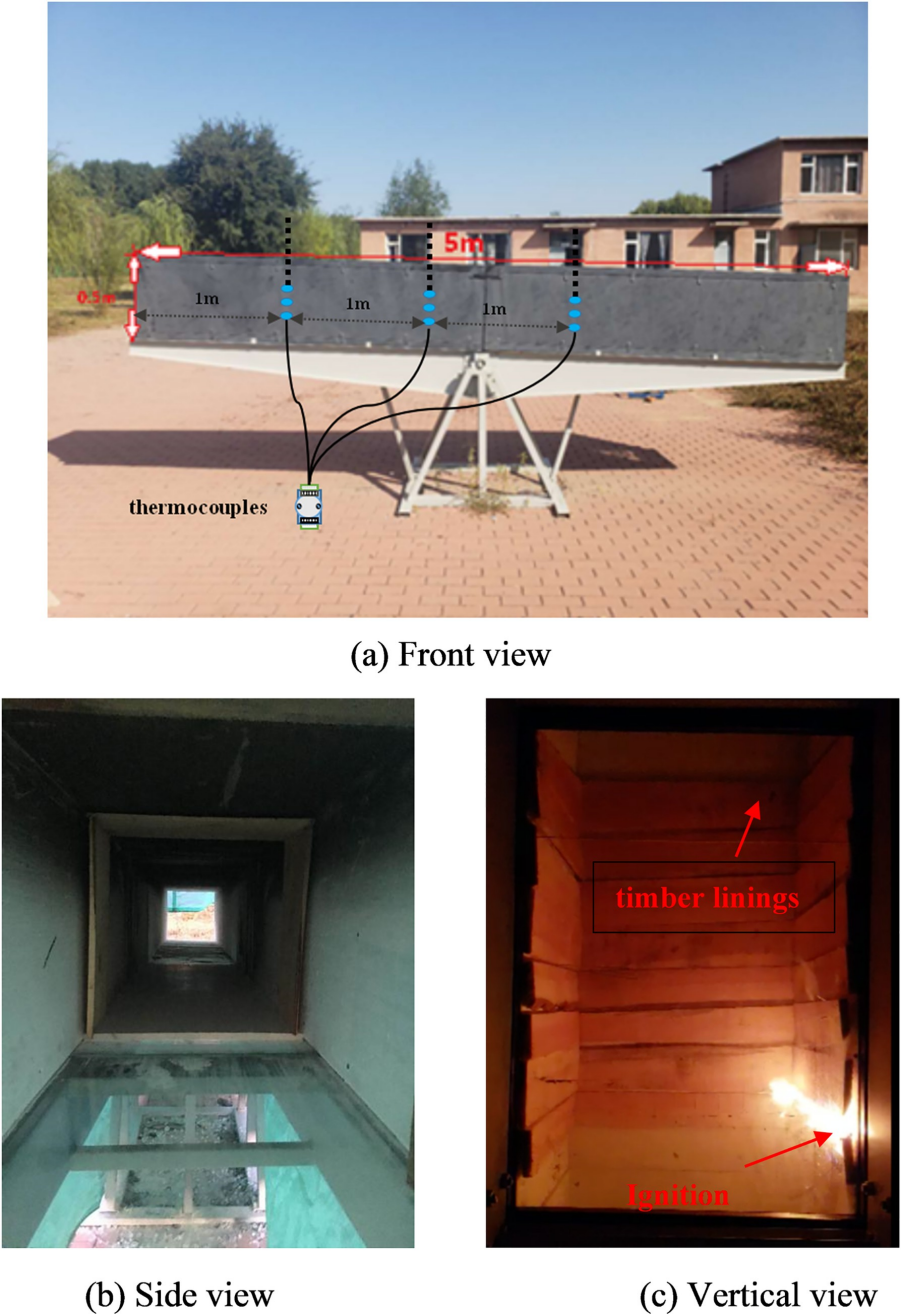
Experimental model.

Fig 1 shows the schematic of the timber linings (0.5 m × 0.5 m × 5.0 m) used for the fire experiments and Fig 2 shows the experimental model. The positions of the thermocouples, camera, and infrared thermal imaging camera (TIC, InfReC R500EX-Pro) are also indicated. The roof and two sides of the experimental tunnel were composed of asbestos boards, and quartz glass was used for the floor. Eight-millimeter-thick pine timber linings with dimensions of 1 m × 0.48 m × 0.48 m were affixed to the roof and two sides 2.5 m away from the right end of the tunnel. N-heptane was used as the ignition source at a distance of 2.5 m from the right end. After the timber linings were ignited at 332°C, the fire source was removed. Nine K-type thermocouples with a 1 mm radius and a measuring range of 0–1300°C were positioned along the wall for measuring the smoke temperature and the depth of the thermocouple in tunnel was 25cm. These thermocouples were grouped into three sets positioned 1.0 m, 2.0 m, and 3.0 m from the inlet opening, each tree was 0.3m,0.2m and 0.1m high from the bottom. The camera (Sony ILCE-5100L APS-C, Pixels:2430, distinguishability: MP4 1440 x1080, Manufacturer: Japan) was set at the right side of the tunnel to record the flame shape in the tunnel. The TIC was also installed on the right side of the tunnel to record the flame structure and temperature, and the transmittance of TIC was set to 0.9.

## 2.2 Fire behaviors observed in experiments

### 2.2.1 Average flame-spread rate

The flame-spread rate was determined by estimating the pyrolysis front on the material surface. For a fire-spread process along only one direction, the average spread rate *V*_p_ can be defined as

$$V_{p}=\frac{X_{p}(t+\Delta t)-X_{p}(t)}{\Delta t}$$ (1)

where *X*_*p*_ *(t)* denotes the pyrolysis front length at time *t*, and *X*_*p*_*(t + Δt)* the pyrolysis front length at time *t + Δt*.

**Fig 3:**
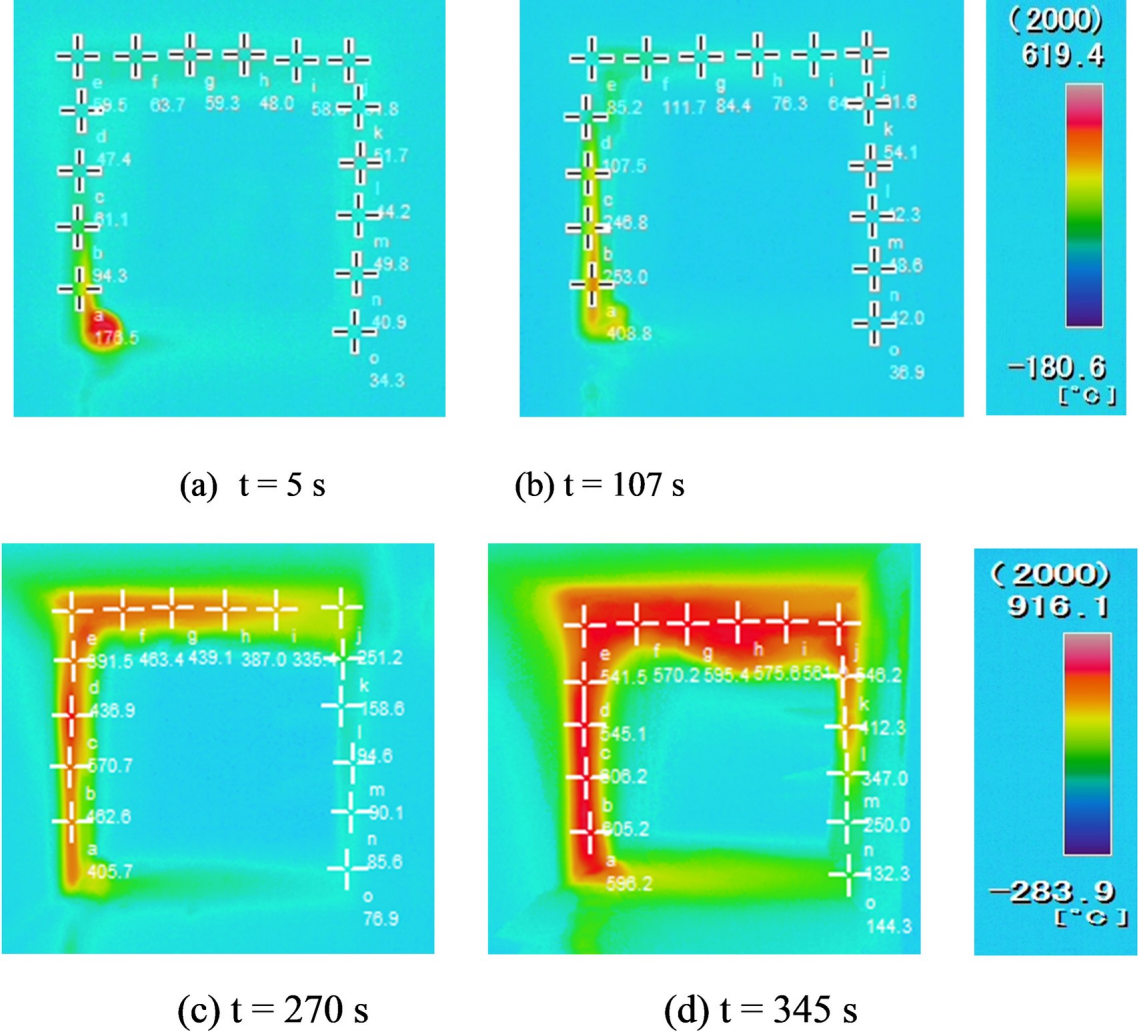
Flame-length changes measured with infrared thermography.

**Fig 4:**
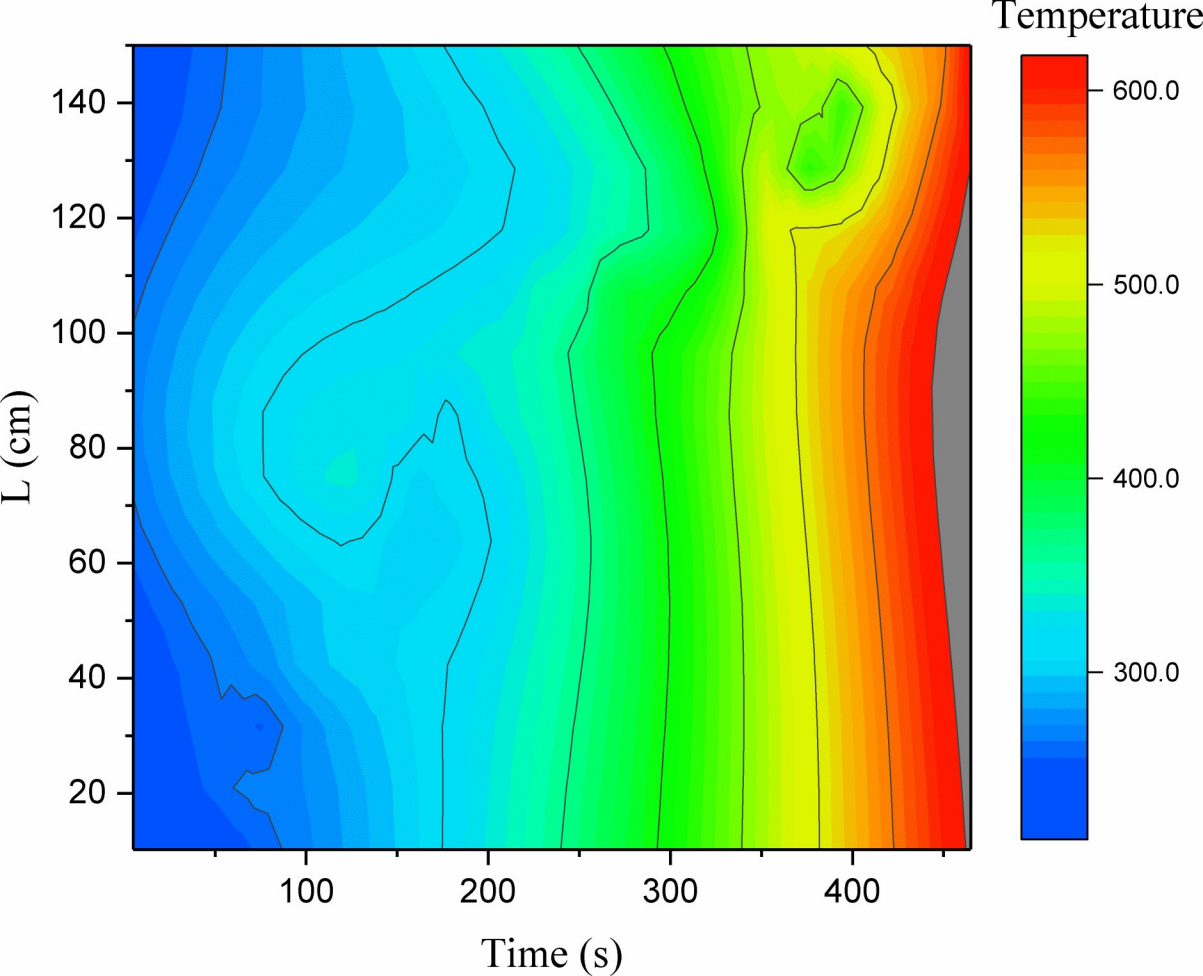
The temperature picture of cross-sectional flame spreading.

**Fig 5:**
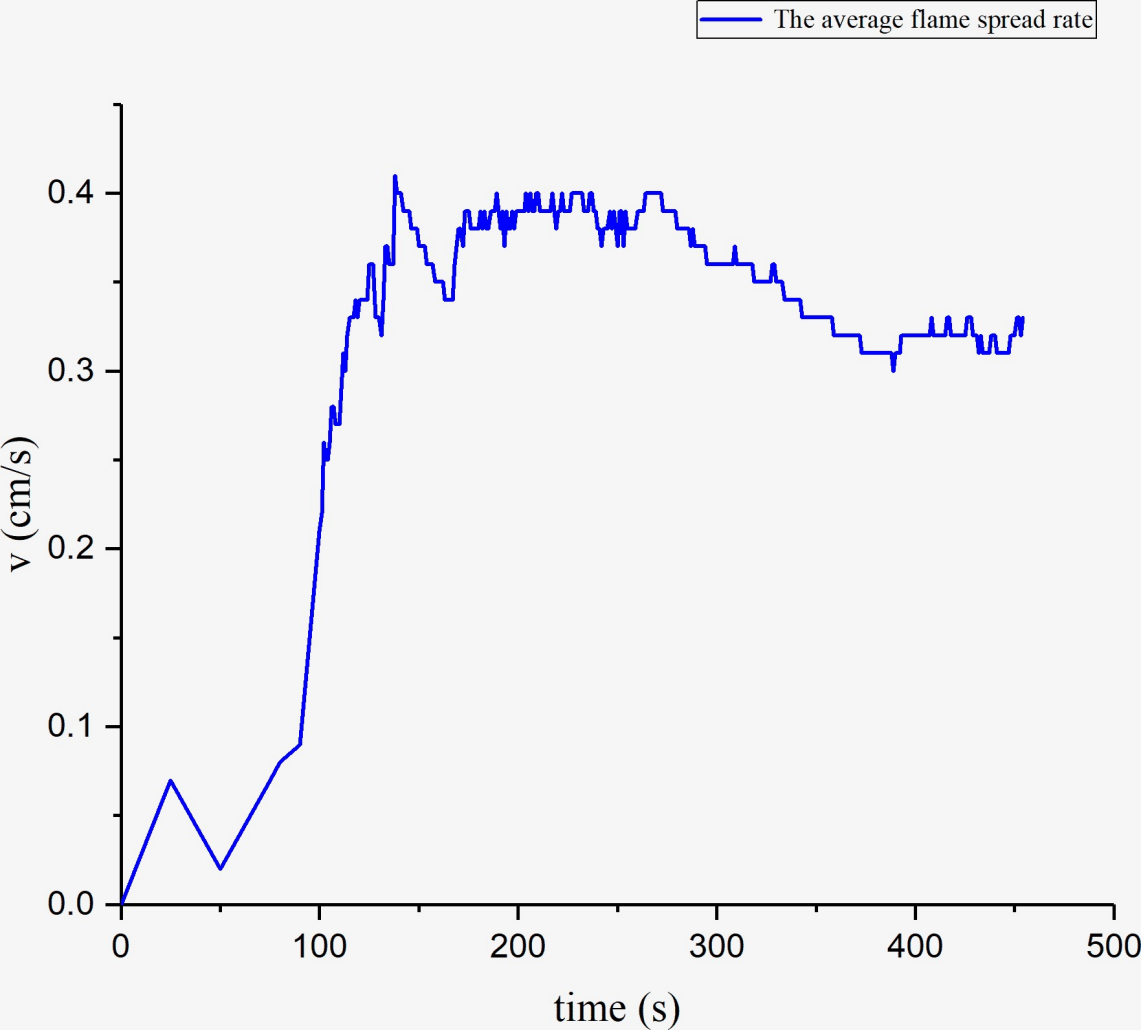
Average cross-sectional flame-spread rate of timber-lining fire.

To get the pyrolysis front length at time, we must determine the pyrolysis temperature first. The infrared thermal imager can be used to record the temperature at any time by appropriately choosing the observation window (Fig 3). We analyzed the characteristics of fire spreading process from temperature, time and space by drawing the temperature picture of cross-sectional flame spreading (Fig 4). The pine temperature was recorded at every 10cm point (Fig 3) with a time interval of 1s, and the temperature picture of cross-sectional flame spreading was drawn. Compared with the temperature color code, the isotherm of the pyrolysis front is marked as light blue, we can conclude that the area below the light blue line is the preheating area and the green area above is the pyrolysis area. While the green area is the pyrolysis scope of pine and the pyrolysis temperature is between 330°C and 480. The pine pyrolysis temperature was set to 420°C in this study, and the pyrolysis front length was estimated as the lining flame length that reached the characteristic pyrolysis temperature. The pyrolysis front length along the cross-section of the timber linings was measured at 1 s intervals. The average flame spread rate over time was calculated using Eq (1). The results are shown in Fig 5.

As shown in Fig 5, the flame-spread rate first decreases and subsequently increases slowly until 100 s, because the timber linings exposed to the air side are heated to decomposition, and the lining interior and exterior are negligibly affected by pyrolysis. However, in the rapid-rise stage (between 100–125 s), the heat penetrates the timber interior through heat conduction, which causes the temperature to rise. Consequently, the pyrolysis products are separated to form fuel vapor on the solid surface. When the fuel concentration in the environment is lower than the surface concentration, the outward fuel vapor transfer is subjected to a concentration gradient. The outward diffusion of fuel on the solid surface and the supply of pyrolysis products within the solid cause the linings to decompose continuously.

It can also be observed from Fig 5 that the average flame-spread rate decreases during the intervals of 125–160 s and 270–380 s because the spread length stagnates when the pyrolysis front reaches the timber-lining corner. When the flame front stagnates, the flame heat is continually transferred to the unburned areas, and therefore, the pyrolysis front continues moving forward. The average flame-spread rate is stable between 160–270 s.

#### 2.2.2 Flame characteristics

**Fig 6:**
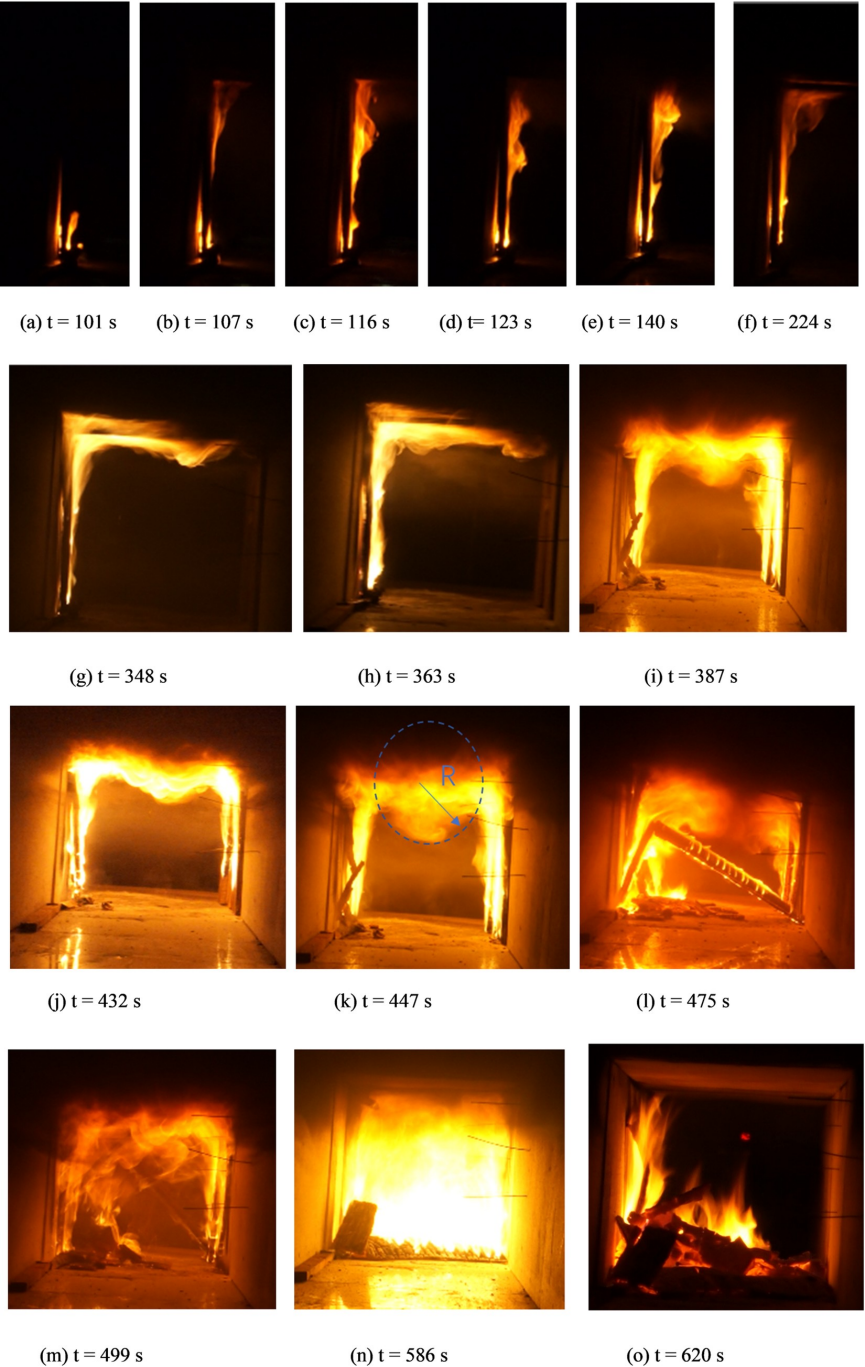
Flame morphology along timber lining during different fire phases.

Fig 6 shows the cross-sectional views of the flame shape of the timber-lining fire in the tunnels at different times. The timber-lining fire process can be divided into four stages: slow rise, rapid rise, stability, and decay. In the slow-rise stage, the flame remains motionless until the lining is ignited (Fig 6A). In the rapid-rise stage, the flame begins to burn vertically along the lining to the ceiling (Fig 6B–6F) and spreads along the timber-lining cross section (Fig 6G–6I). In the stability stage, the flame spreads along the longitudinal lining direction, and the fire reaches the full-combustion mode (Fig 6J–6N). When the amount of combustible material gradually decreases, the fire enters the decay stage (Fig 6O).

From Fig 6B, it can be observed that the fire source is close to the timber linings, which are ignited at 101 s. At this time, air may be entrained from the open side, and the flame “clings” to the linings, which can greatly intensify the fire and cause rapid spreading. The flame on the roof of the tunnel linings forms at around 224 s, as shown in Fig 6F. At 348 s [Fig 6G], the ceiling flame develops further, with the flame length increasing and the vertical-fire-plume diameter gradually increasing. The plume is roughly semicircular in the impact zone of the ceiling, and the smoke layer leaving the edge of the impact zone is not very thick. Moreover, the flame at the ceiling is diffused. This is because the flame travels downward under the constraint of the sidewall and ceiling, whereas the flame is aided by the vertical upward buoyancy. The flame eventually reaches a stationary point and travels in the opposite direction to form a vortex structure [Fig 6I–6K]. During this process, the flame entrains more air than a horizontal spreading flame. With continuous combustion, the flame length, smoke-flow thickness, and vertical-fire-plume diameter increase significantly between 344 s and 363 s, and the fire scale develops to a significant extent. From Fig 6I–6K, it can be observed that the flame spreads to the other side at 387 s, the fire plumes of the two lining sides entrain air from the open side, and the flame spreads upward along the vertical linings. Subsequently, the vertical flame impacts the ceiling to form a large-scale vortex. This is because the smoke flow, which is blocked by the side linings, forms a counter-buoyancy jet in the process of the longitudinal fire spread, causing part of the smoke to “return.” The two sides of the impacted plume form “M” shapes. As the flame spreads continually, it tends to move downward. At 475 s [Fig 6L], with the flame temperature rising, the timber linings begin to collapse, and a disordered smoke flow occurs. At 499 s [Fig 6M], axisymmetric fire plumes appear on both sides with the fire plume absorbing cold air continuously, and the tunnel cross-section is almost entirely covered by flames. After 620 s, the flame decays gradually, and the fire is extinguished.

#### 2.2.3 Temperature distribution

**Fig 7:**
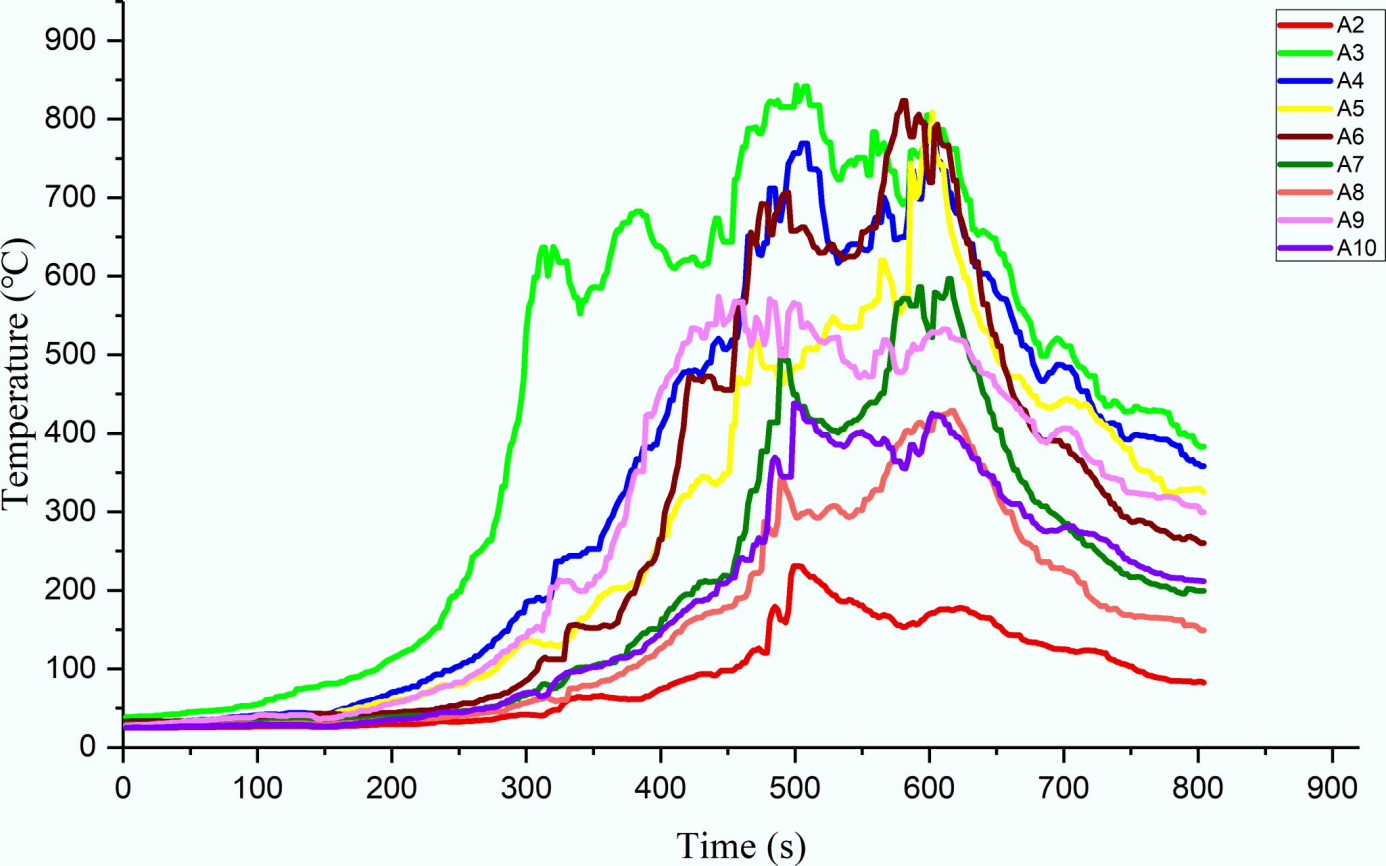
Smoke-temperature variation in the tunnel.

In tunnel fires, the high temperature above and around the fire source often causes significant damage to the tunnel structure. Thus, it is necessary to first determine the smoke temperature in tunnel fires. Fig 7 shows the smoke-temperature variation measured with the use of a series of thermocouples positioned under the ceiling.

From Fig 7, it can be observed that the temperature curves can be classified into four stages: slow rise, rapid rise, stability, and decline. When the fire is close to the lining, the flame temperature at each measuring point increases slowly in the initial fire stage. The temperature increases sharply after 200 s. Meanwhile, the temperature of the sidewall increases owing to the continuous combustion of the fire source, and the thermal feedback from the sidewall to the fire source increases accordingly, causing the maximum temperature under the lining ceiling to rise. As shown in Fig 7, the maximum flame temperature at each measuring point is 800–900°C, and it can be observed that the high-temperature flame area moves slowly toward both sides of the tunnel.

Upon comparing the three thermocouple datasets (A2–A4, A5–A7, and A8–A10) in Fig 7, it can be found that the “arrival” times corresponding to the maximum temperatures at A2, A3, and A4 are reached earlier than those at other measuring points at the same height. This is because it takes time for the flame front to reach a location far from the fire source, resulting in a delay in the temperature rise.

## 3. Numerical simulation model and experimental validation

### 3.1 Brief description of simulation settings

The fire dynamics simulator (FDS), based on numerical solutions of the Navier–Stokes equations, was employed in this work to analyze timber-lining fires occurring in a tube. Fire Dynamics Simulator (FDS) has been widely used in research of fire behavior and its validity has been extensively verified [28, 29]. FDS solves numerically a form of the Navier–Stokes equations for thermally-driven flow. A description of the model, many validation examples, and a bibliography of related papers and reports may be found on the homepage of FDS. It includes both DNS (Direct Numerical Simulation) model and LES (Large Eddy Simulation) model. The LES model is mainly applied in simulating fire smoke flow process in large space, which is widely used in study of fire-induced smoke flow behavior, is selected in this study [30]. The distribution of temperature in the tube was measured for the fire spread in the simulations.

The heat transfer and pyrolysis inside the timber linings are modelled. The model describes the conduction of heat inside the material, the evaporation of moisture and the degradation of virgin material to gaseous fuel and char. The volatile gases are instantaneously released to the gas space. The governing equation for energy, mass conservation and decomposition equations are as follows:

$$\bar{\rho c}\frac{\partial T}{\partial t}=\frac{\partial }{\partial x}(k_{s}\frac{\partial T}{\partial x})+\frac{\partial \rho_{s}}{\partial t}[\Delta H_{py}-C(T-T_{0})]+\frac{\partial \rho_{m}}{\partial t}[\Delta H_{ev}-D(T-T_{0})]$$ (2)

$$\frac{\partial \rho }{\partial t}=-A(\rho -\delta )e^{-E_{A}/RT}$$ (3)

$$\frac{\partial m}{\partial x}=\frac{\partial \rho }{\partial t}$$ (4)

Where *ρ*_*s*_ is the total density of the solid, and *ρ*_*m*_ is the moisture density. The boundary condition on the surface is due to the convection and radiation

$$-k_{s}{\frac{\partial T}{\partial x}|}_{solid}={\overset{˙}{q}}_{rad}^{\prime \prime }-k_{gas}{\frac{\partial T}{\partial x}|}_{gas}$$ (5)

Where ${\overset{˙}{q}}_{rad}^{\prime \prime }$ is the net radiative heat flux on the surface, *k*_*s*_ and *k*_*gas*_ are the solid and gas phase conductivities, respectively. The back side boundary condition is either adiabatic or convection to the back side gas. *ΔH*_*py*_ and *ΔH*_*ev*_ are the heat of pyrolysis and the heat of water evaporation. Coefficient C and D are defined as

$$C=\frac{\rho_{S0}{\bar{c}}_{p,s0}-\rho_{char}{\bar{c}}_{p,char}}{\rho_{s,0}-\rho_{char}}-{\bar{c}}_{p,g}$$ (6)

$$D={\bar{c}}_{p,m}-{\bar{c}}_{p,g}$$ (7)

Where $\rho_{S0},\;{\bar{c}}_{p,s0},\;\rho_{char}$ and ${\bar{c}}_{p,char}$ are the densities and specific heats of the virgin material and char, respectively, and ${\bar{c}}_{p,g}$ and ${\bar{c}}_{p,m}$ are the specific heats of gaseous products and moisture. The overbars in Eqs (4) and (5) denote the average of values at instantaneous temperature *T* and initial temperature *T*_*0*_. The pyrolysis rate of the material is modelled as a first order Arrhenius reaction

$${\overset{˙}{m}}^{\prime \prime }=Ae^{-E_{A}/RT}$$ (8)

Where *A* is the pre-exponential factor and *E*_*A*_ is the activation energy. The coefficients *A* and *E*_*A*_ are chosen such that the pyrolysis takes place close to a given pyrolysis temperature. The modelling reported in this text was done using a pre-release version of FDS 6, which employs Eq (5) to describe the pyrolysis rate. The forthcoming official release version of the FDS 6 will, However, use a slightly different formulation of the pyrolysis rate, with the densities of the virgin material and char expressed explicitly:

$${\overset{˙}{m}}^{\prime \prime }=A(\rho_{s0}-\rho_{char})e^{-E_{A}/RT}$$ (9)

This formulation is closer to the traditional form of the pyrolysis equations and it also makes the coefficient *A* less dependent of the material density.

The following definitions are used to calculate the thermal properties of the material during the drying and charring processes.


$$\bar{\rho c}=(\rho_{a}c_{p,s0}+\rho_{c}c_{p,c}+\rho_{m}c_{p,m})\;k_{s}=k_{s0}(\frac{\rho_{a}}{\rho_{s0}})+k_{char}(\frac{\rho_{c}}{\rho_{char}})$$ (10)

$$\rho_{a}=\rho_{s0}\frac{\rho_{s}-\rho_{char}}{\rho_{s0}-\rho_{char}}\rho_{c}=\rho_{s}-\rho_{a}$$ (11)

**Fig 8:**
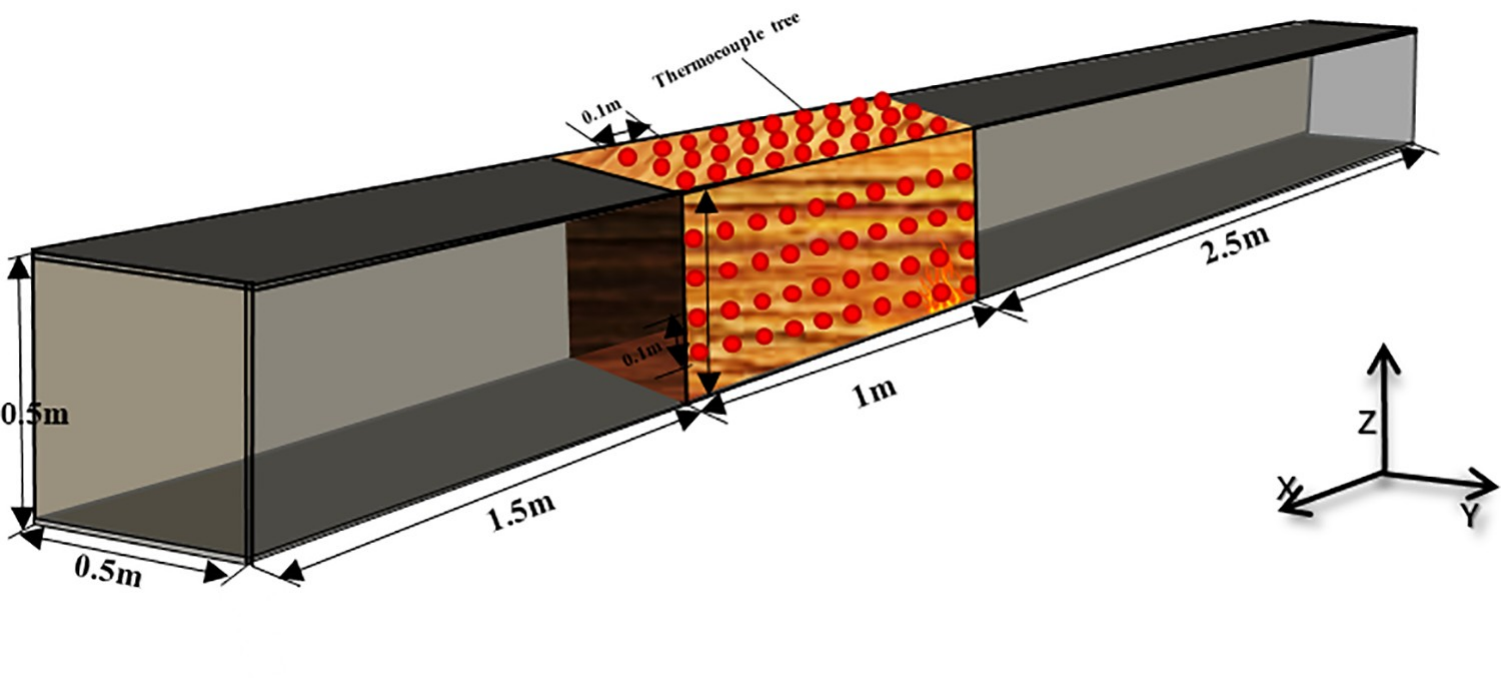
Physical model and the arrangement of thermocouples.

A horizontal tube was used as the simulation model. The length of this horizontal “tunnel” was fixed at 5 m, and the cross-sectional dimensions were 0.5 m (width) × 0.5 m (height), as shown in Fig 8. The outside wall was composed of steel, and the sidewalls were constructed using 5 mm-thick asbestos boards for thermal insulation. A set of 8 mm-thick pine timber linings with dimensions of 1 m × 0.5 m × 0.4 m was affixed at the tunnel top and sides 1.5 m away from the left end. The fuel of N- heptane was ignited close to the sidewall and 2.5 m away from the right end. The heat release rate of the fire source can be calculated for heptane fires by the following equation:

$$\overset{˙}{Q}=\chi_{c}{\overset{˙}{m}}^{\prime \prime }A_{f}\Delta H_{c}$$ (12)

Here, $\overset{˙}{Q}$ denotes the heat release rate (kW), *χ*_*c*_ the combustion efficiency (<1), ${\overset{˙}{m}}^{\prime \prime }$ the mass loss flux (kg/m^2^·s), *A*_*f*_ the surface area of fuel (m^2^), *ΔH*_*c*_ the heat of combustion of N- heptane (KJ/kg). Based on the equation, 1 kg of heptane combustion could produce 48.17 ×10^3^KJ heat, while the mass loss flux of N-heptane with a diameter of 0.17m in the Stable phase is 0.017 kg/m^2^·s [31], we can calculate the heat fluxes of fire source is 6 kW. The heat flux divided by the fire area is the heat release rate per unit volume 800 kW/m^2^.

The parameters of combustion and flame spreading behaviors of timber linings are shown in Table 1. The air density and gravitational acceleration are evaluated at a standard atmospheric pressure. The ambient temperature is 20°C measured by the thermometer. The solid density and combustion heat of pine are 640 kg/m^2^ and 1.64×10^4^ kJ/kg, while the conductivity and flame emissivity of yellow pine are set as 0.14W/(m·k) and 0.9 respectively [32]. The ignition temperature of pine evaluated by using cone calorimeter is 332°C [33]. The moisture content of pine is measured with Drying method and calculated by the formula $W=\frac{G_{s}-G_{go}}{G_{s}}\times 100\%$ (*G*_*s*_ denotes the pine sample weight, *G*_*go*_ the drying weight).

**Table 1:** Parameters related to combustion and flame-spreading behaviors of timber linings.

Parameters	Value	Parameters	Value
Air density (kg/m^3^)	1.293	Ignition temperature (°C)	332
Ambient pressure (kPa)	101.3	Heat of combustion(kJ/kg)	1.64×10^4^
Acceleration due to gravity (m/s^2^)	9.8	Conductive and radiative heat fluxes (kW)	6
moisture content (%)	16.2	Heat release rate per unit volume (kW/m^2^)	800
Ambient temperature (°C)	20	conductivity W/(m∙k)	0.14
Solid density(kg/m^2^)	640	flame emissivity	0.9

In this paper, a set of simulations with FDS was conducted to investigate flame spread of timber-linings in a longitudinal tunnel. In the simulations, the smoke temperature was measured by nine thermocouples that were grouped into three sets positioned 1.0 m, 2.0 m, and 3.0 m from the inlet opening. In addition, 15 thermocouple trees with 10 thermocouples per tree were positioned to measure the air temperature around the timber linings contributing to the flame spread. These were firmly affixed to the lining surface at 0.1 m intervals (Table 2). Each row was numbered 1–10 from left to right. The thermocouples were inserted into the linings at an insertion depth of 1 cm. Finally, all the linings with thermocouples were selected to monitor the temperature variation in the timber-lining fire.

**Table 2:** Layout of thermocouple trees positioned on the lining inner surface (unit: m).

Measurement points		Interval distance (m)	Height (m)	Distance from the ignition surface (m)
sidewall Y = 0.02 m	A1–A10	0.1	0.1	0.03
	B1–B10	0.1	0.2	0.03
	C1–C10	0.1	0.3	0.03
	D1–D10	0.1	0.4	0.03
	E1–E10	0.1	0.48	0.03
sidewall Z = 0.48 m	F1–F10	0.1	0.48	0.06
	G1–G10	0.1	0.48	0.15
	H1–H10	0.1	0.48	0.24
	I1–I10	0.1	0.48	0.33
	J1–J10	0.1	0.48	0.42
sidewall Y = 0.48 m	K1–K10	0.1	0.48	0.47
	L1–L10	0.1	0.4	0.47
	M1–M10	0.1	0.3	0.47
	N1–N10	0.1	0.2	0.47
	O1–O10	0.1	0.1	0.47

Various simulations scenarios were implemented to study the influence of different wind speeds on the flame spread and smoke movement related to the timber-lining fire. In the mining, the wind speeds at the entrance were 0.25m/s-6m/s. While the fire was extinguished at 1m/s due to the scale limited in this simulation. Therefore, this paper focused the wind speed range from 0 to 0.75m/s. Thus, wind speeds of 0 m/s, 0.25 m/s, 0.5 m/s and 0.75 m/s were considered.

### 3.2 Sensitivity study of the grid system

In FDS simulations, the grid size is a key parameter. The A*D*/*δ*_*x*_ criterion has been widely used for assessing the grid resolution, where *δ*_*x*_ denotes the grid size, and the characteristic length D* is calculated as

$$D^{*}={\left(\frac{\overset{\cdot }{Q}}{\rho_{\infty }c_{p}T_{\infty }\sqrt{g}}\right)}^{\frac{2}{5}}$$ (13)

Here, D* represents the dimensionless grid characteristic length, $\dot{Q}$ the heat release rate (kW), *ρ*_*∞*_ the density of ambient air (kg/m^3^), *c*_*p*_ the specific heat of air at constant pressure [kJ/(kg·K)], *T*_*∞*_ the ambient air temperature (K), and *g* the acceleration due to gravity (m/s^2^).

Following McGrattan et al. [31], the value of D*/δ_*x*_ was set to lie in the range of 4 to 16. Next, the grid size of the finest mesh for a 6kW fire was calculated to lie between 0.02 m and 0.48 m. Obviously, a finer grid will better reflect the heat flow field in terms of detail; however, this makes the simulation time consuming. Therefore, it is necessary to choose an appropriate mesh grid size. In this work, a multi-mesh system with grid size of 0.02 m (*δ*_*x*_) * 0.02 m (*δ*_*y*_) * 0.025 m (*δ*_*z*_) was selected.

### 3.3 Verification of numerical model

**Fig 9:**
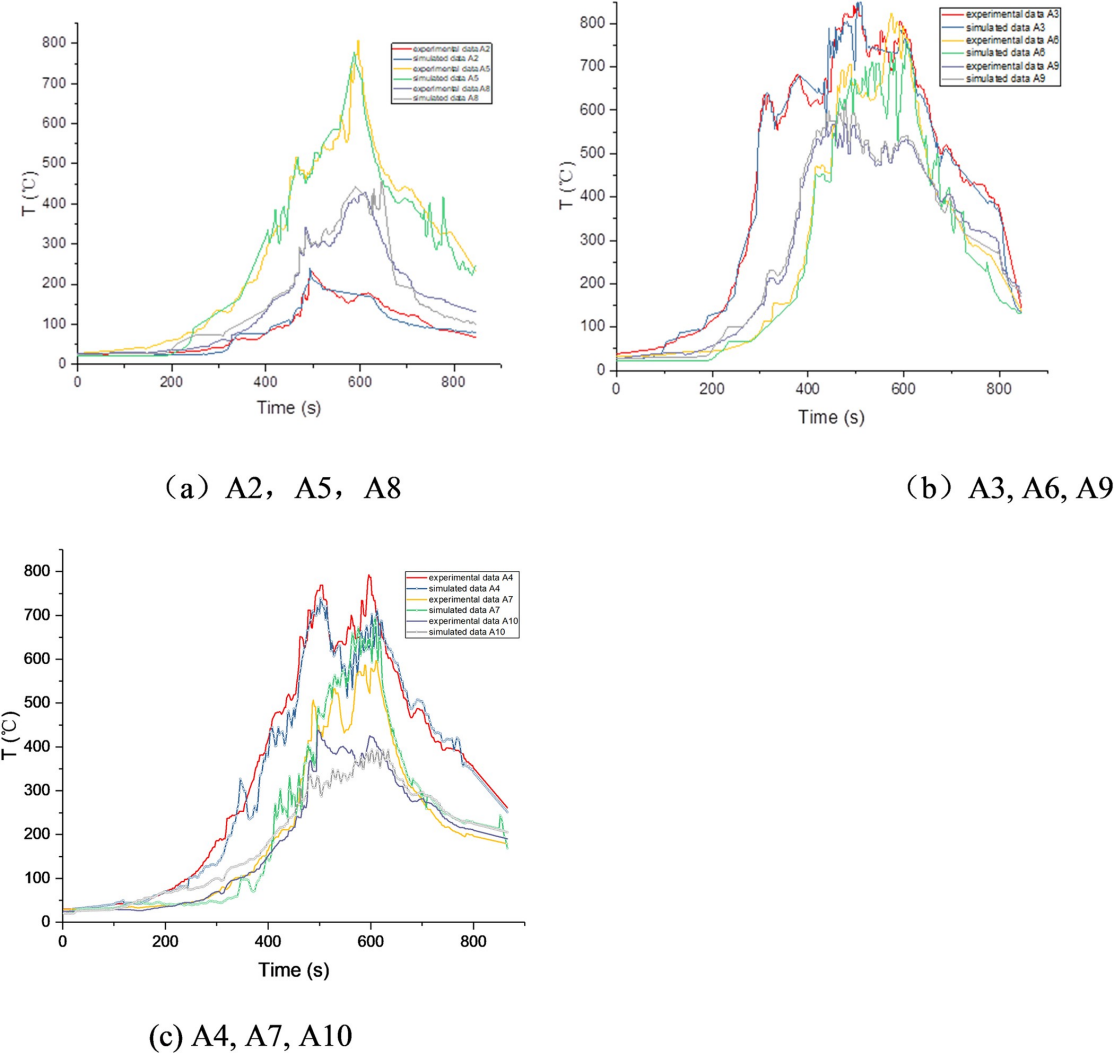
Comparison between experimental and simulated smoke temperatures.

To confirm the validity of the numerical simulations, we chose the representative thermocouples A2~A10 to compare the simulated smoke temperature with the experimental result (Fig 9). The figure below shows the experimental and simulated smoke-temperature results. It can be observed that the simulated data are fairly consistent with the experimental data in the slow-rise stage and rising stage; however, there are some deviations between the simulated and experimental data in the stable-combustion stages, which is caused by the smoke layer above the tunnel. In the stable-combustion stage, the maximum deviation between the two curves ranges from 30–50°C. The highest simulated temperature under the lining ceiling ranges between 750 and 850°C, which is consistent with the experimental temperature. The relative error between the experimental and simulated data is within 6%.

**Fig 10:**
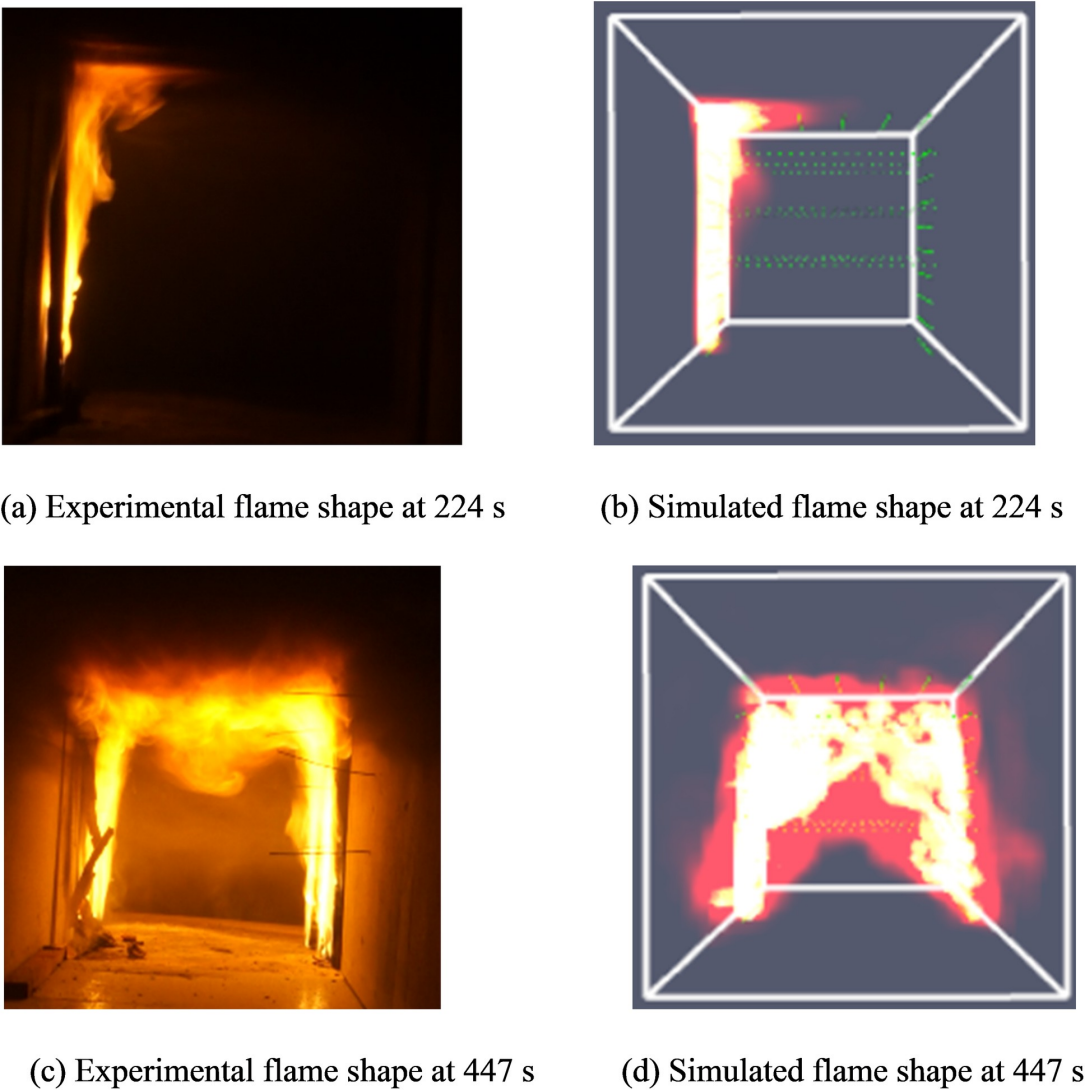
Comparison of the flame shape cross-section between experiments and fire simulations.

Fig 10 shows the cross-sectional flame shape obtained as per experiments and fire simulation results. It can be observed that the simulated flame shape is fairly consistent with the experimental flame shape in the rising and stable stages. Thus, the numerical simulations conducted in the study can be considered valid.

## 4. Simulation results and discussion

The process underlying timber-lining fires is complex, and moreover, such fires are strongly affected by the ventilation flow. The flue gas and heat generated in the fire process cannot easily be cleared (or vented), and they spread quickly across the entire tunnel; therefore, it is necessary to study the effect of ventilation flow on fire. The fire behavior of timber linings under different wind speeds in the tunnel is discussed through experiments and simulations.

### 4.1 Flame behavior and spread process

**Fig 11:**
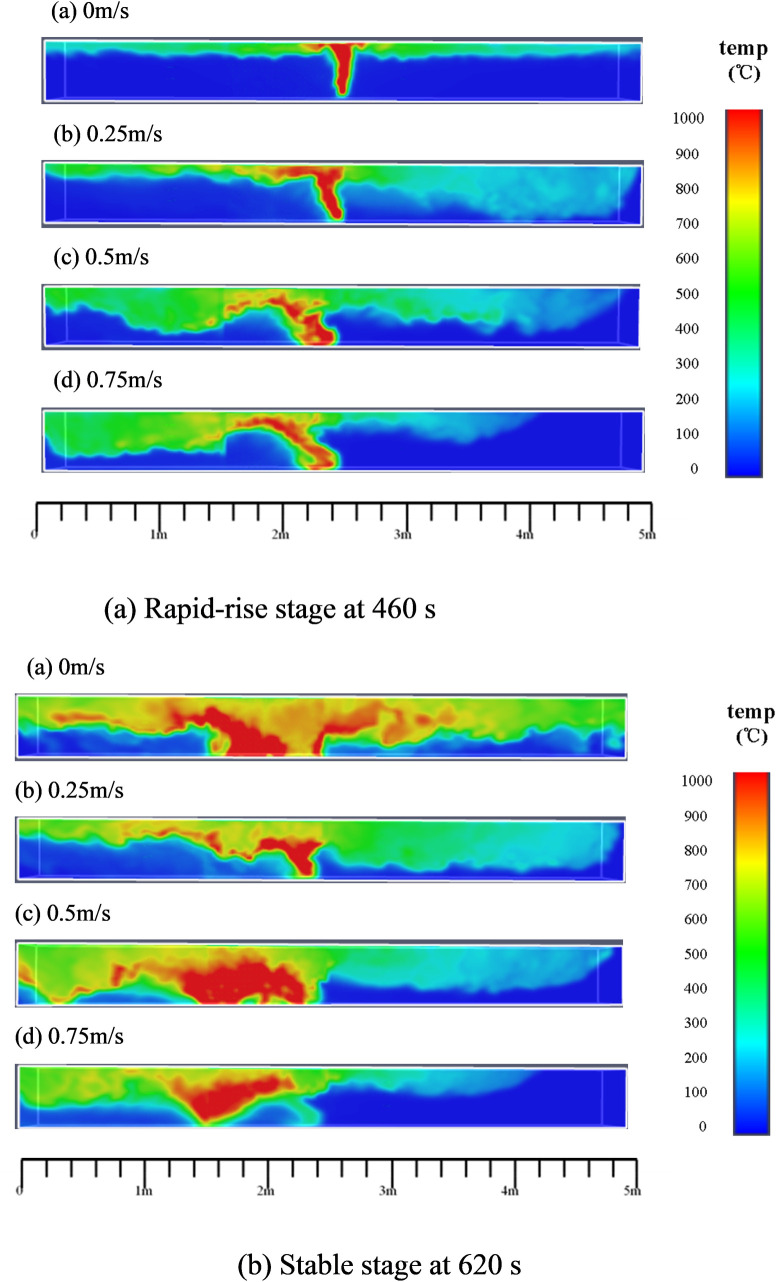
Temperature field of timber-lining fire at different wind speeds (Y = 0.02 m).

**Fig 12:**
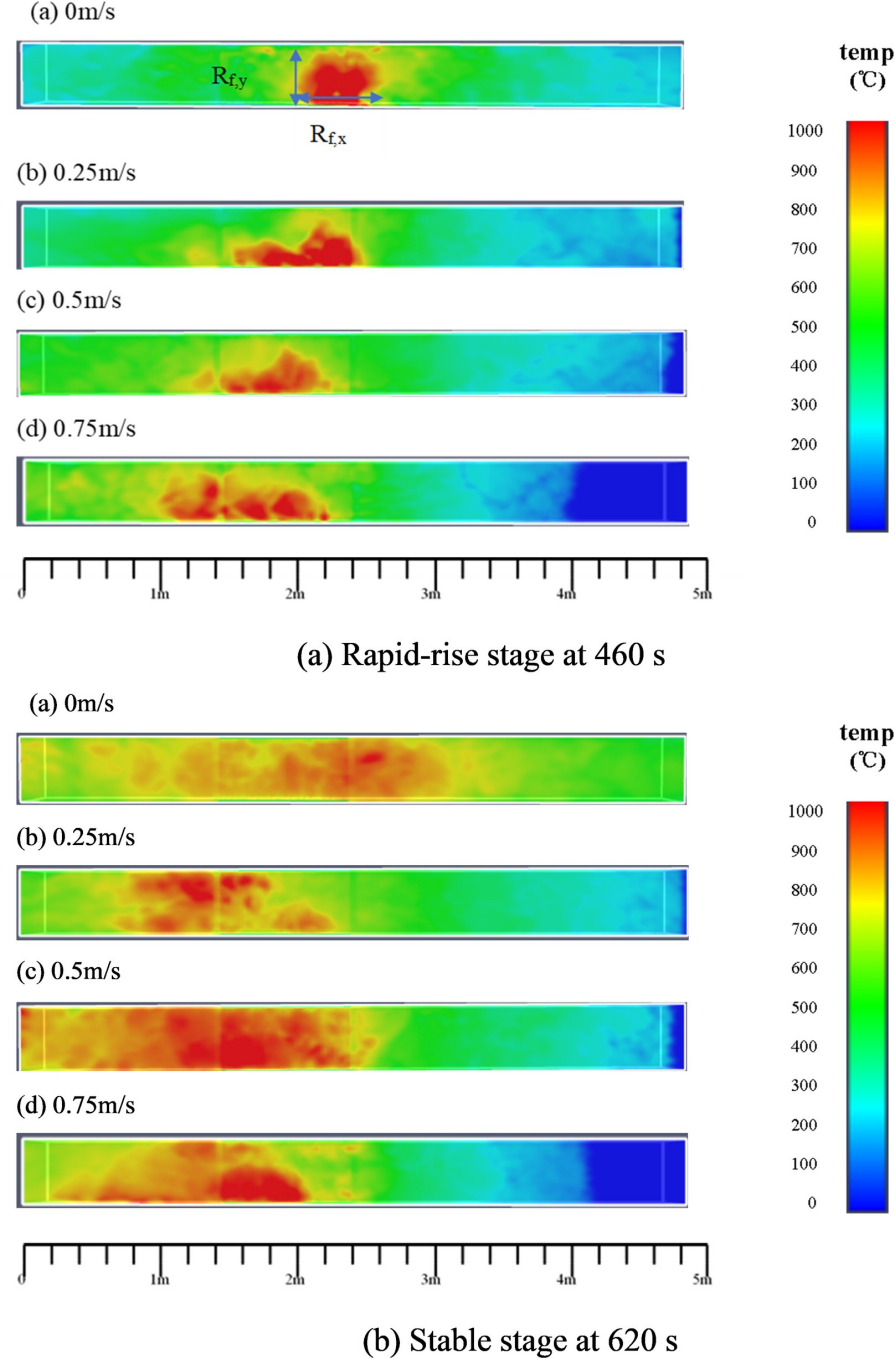
Ceiling temperature field of timber-lining fire at different wind speeds (z = 0.48 m).

**Fig 13:**
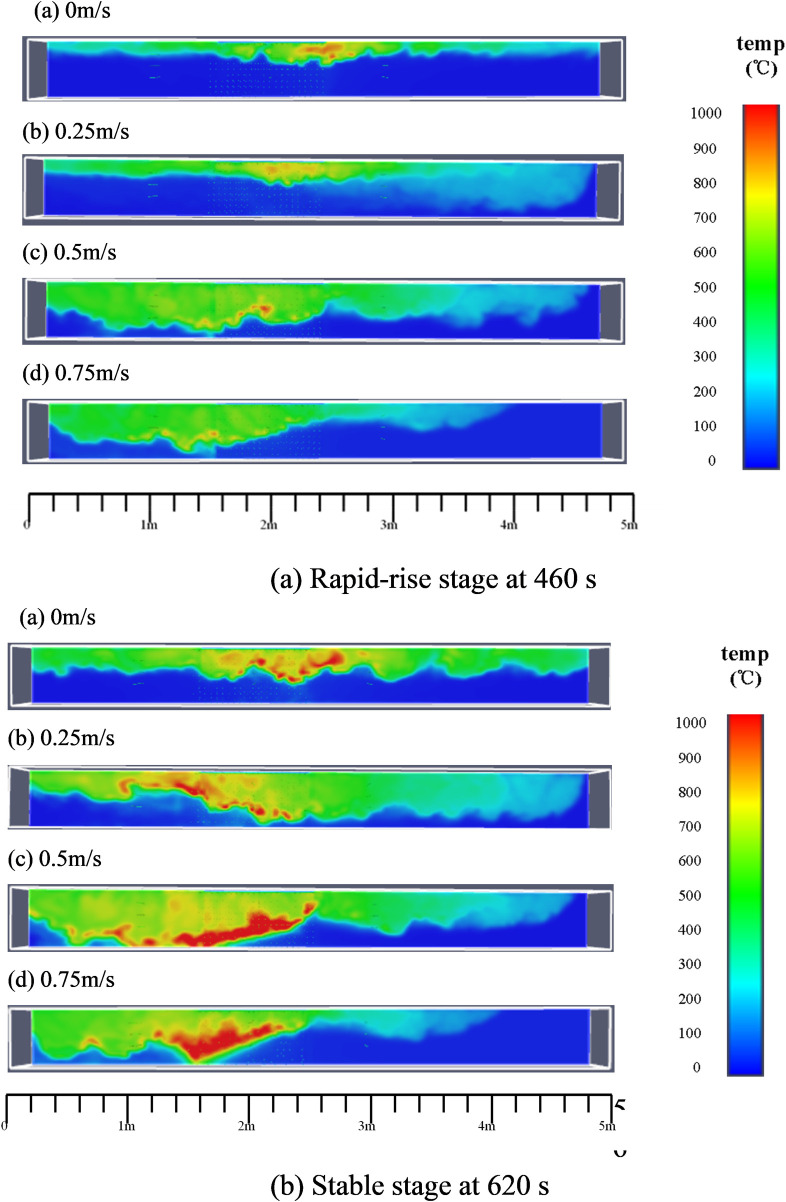
Temperature field of timber-lining fire at different wind speeds (Y = 0.48 m).

Figs 11–13 show the three-dimensional temperature field (Y = 0.02 m, Z = 0.48 m, and Y = 0.48 m, respectively) for different wind speeds. For Y = 0.02 m (Fig 11), it can be observed that the flame is perpendicular to the fire source at 460 s under quiescent conditions. However, as the wind speed increases to 0.25 m/s, 0.5 m/s, and 0.75 m/s, the flame gradually deflects away from the vertical direction. Greater wind speeds correspond to greater deflection angles of the combustion flame.

**Fig 14:**
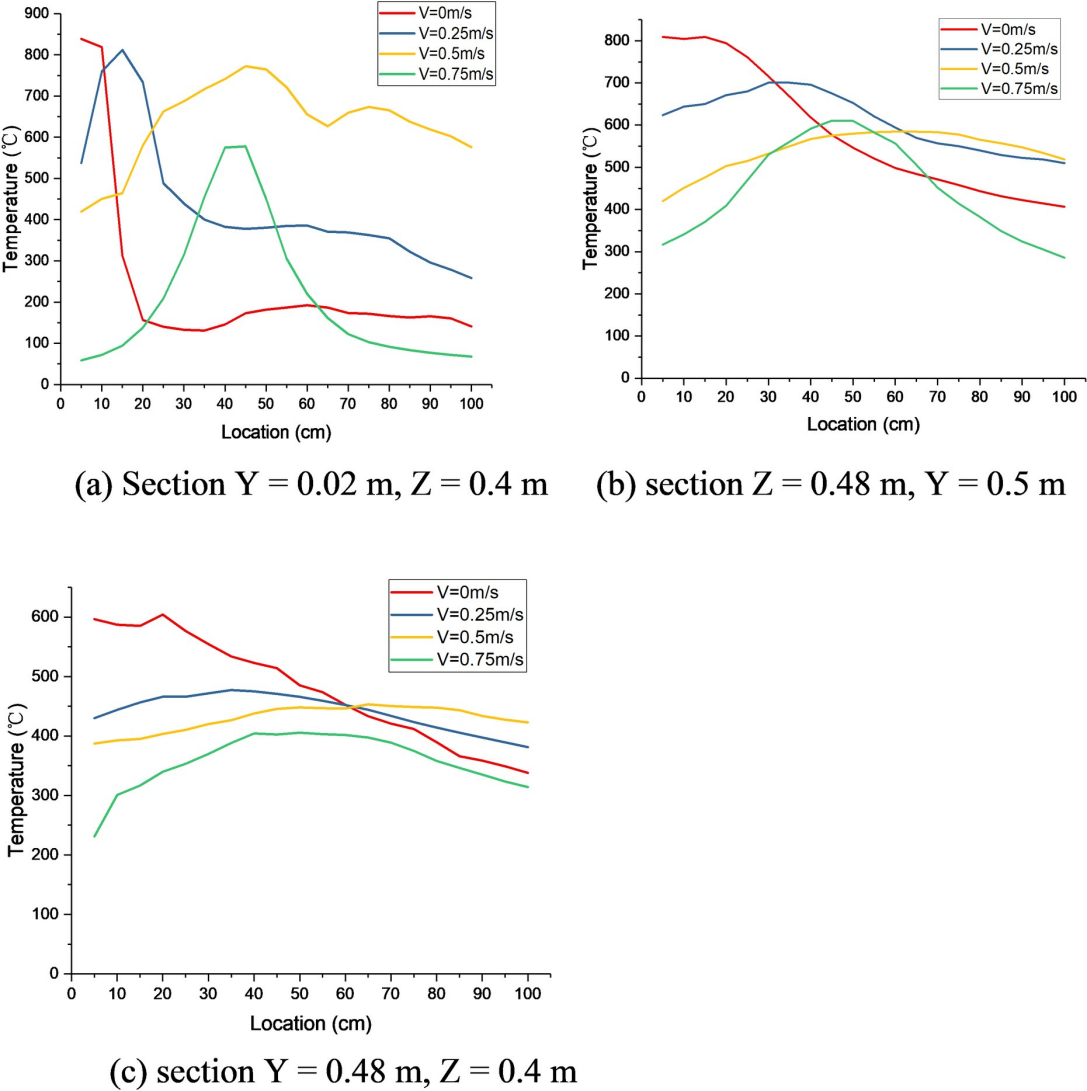
Horizontal temperature curves of timber linings at 460 s in the tunnel for different wind speeds.

From Figs 11–13, it can be observed that the flame follows the sidewall-ceiling-sidewall “pathway” along the cross section in the rapid-rise stage, and subsequently, the flame extends gradually to both lining ends along the three dimensions of the timber linings in the stable stage. As mentioned earlier, thermocouples were used to measure the lining temperatures to study the heat transfer and fire spread processes. Fig 14 shows the temperature curves of the timber linings at 460 s in the tunnel for different wind speeds. Under quiescent conditions, it can be observed that the maximum temperature in the three sections is first recorded in the tunnel cross-section vertically above the fire source, and subsequently, the temperature decreases gradually from the fire source to both ends of the walls. With the increase in the wind speed, the maximum temperature in the three sections is first recorded in the tunnel cross-section farther away from the fire source along the wind direction. Higher wind speeds corresponds to greater distances of the maximum-temperature points of the three sections from the fire source.

### 4.2 Flame length

**Fig 15:**
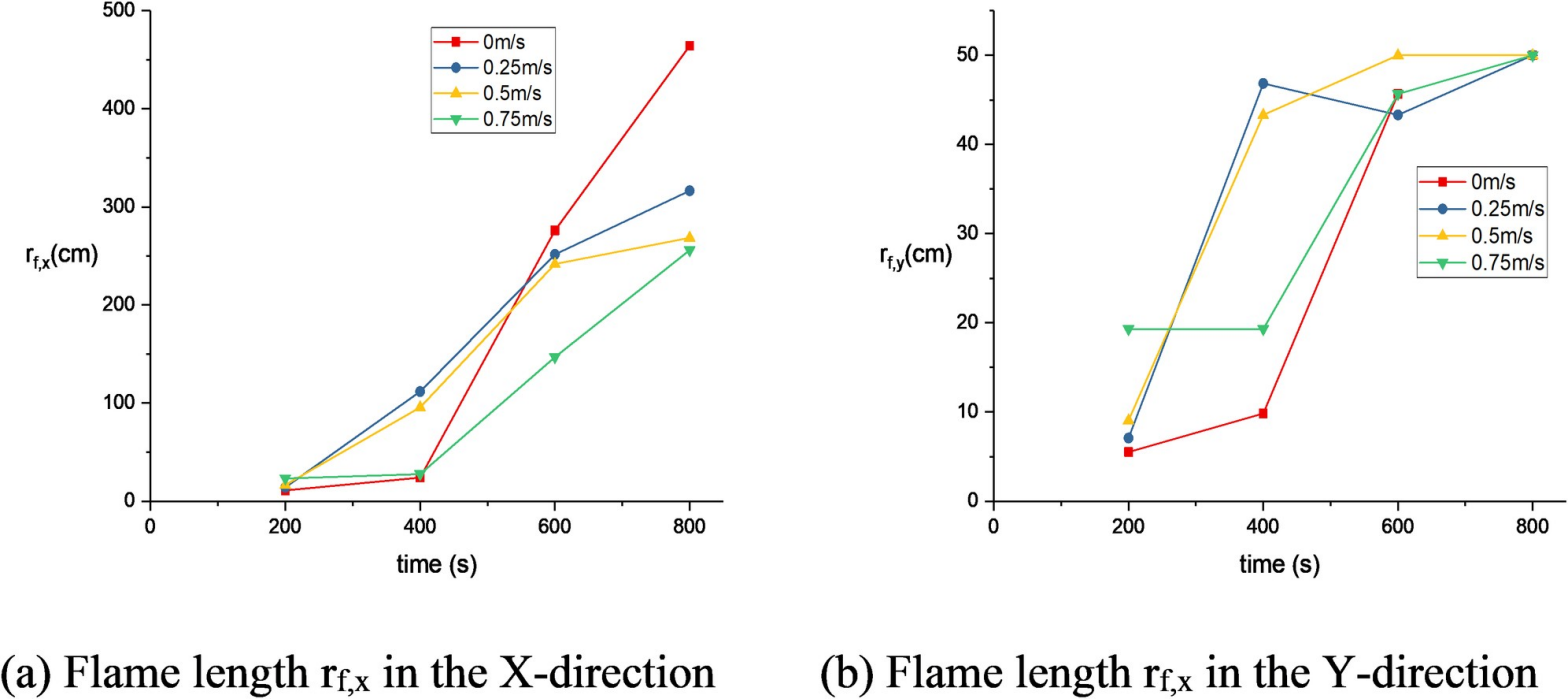
Flame lengths (r_f_) underneath the ceiling in the x- and y-directions for different wind speeds.

**Fig 16:**
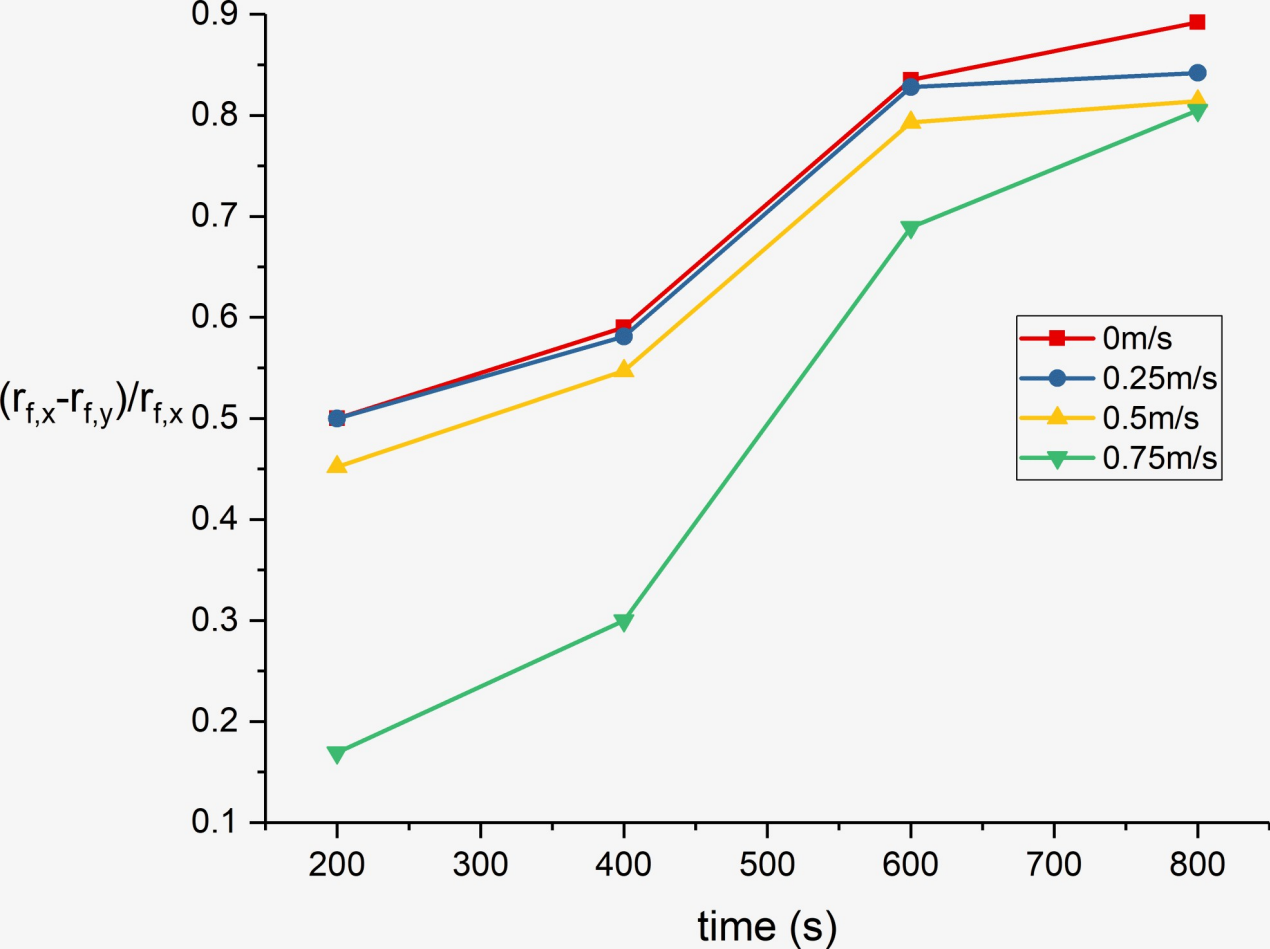
Normalized flame lengths *R*(*f*) in the x- and y-directions for different wind speeds.

The flame lengths on the ceiling were estimated using an averaging method to obtain the flame appearance probability contour, as shown in Fig 12A. Fig 15 shows the exact flame length underneath the ceiling in the x- and y-directions for different wind speeds. Fig 16 presents the normalized flame lengths *R*(*f*) = (*r*_*f*,*x*_−*r*_*f*,*y*_)/*r*_*f*,*x*_ in the two directions for different wind speeds.

It can be observed that *R*(*f*) significantly decreases with increase in the wind speed. This is because the air flow causes high-temperature smoke flow along the longitudinal section, and the flame length in the x-direction is restricted by the air flow.

### 4.3 Smoke temperature

**Fig 17:**
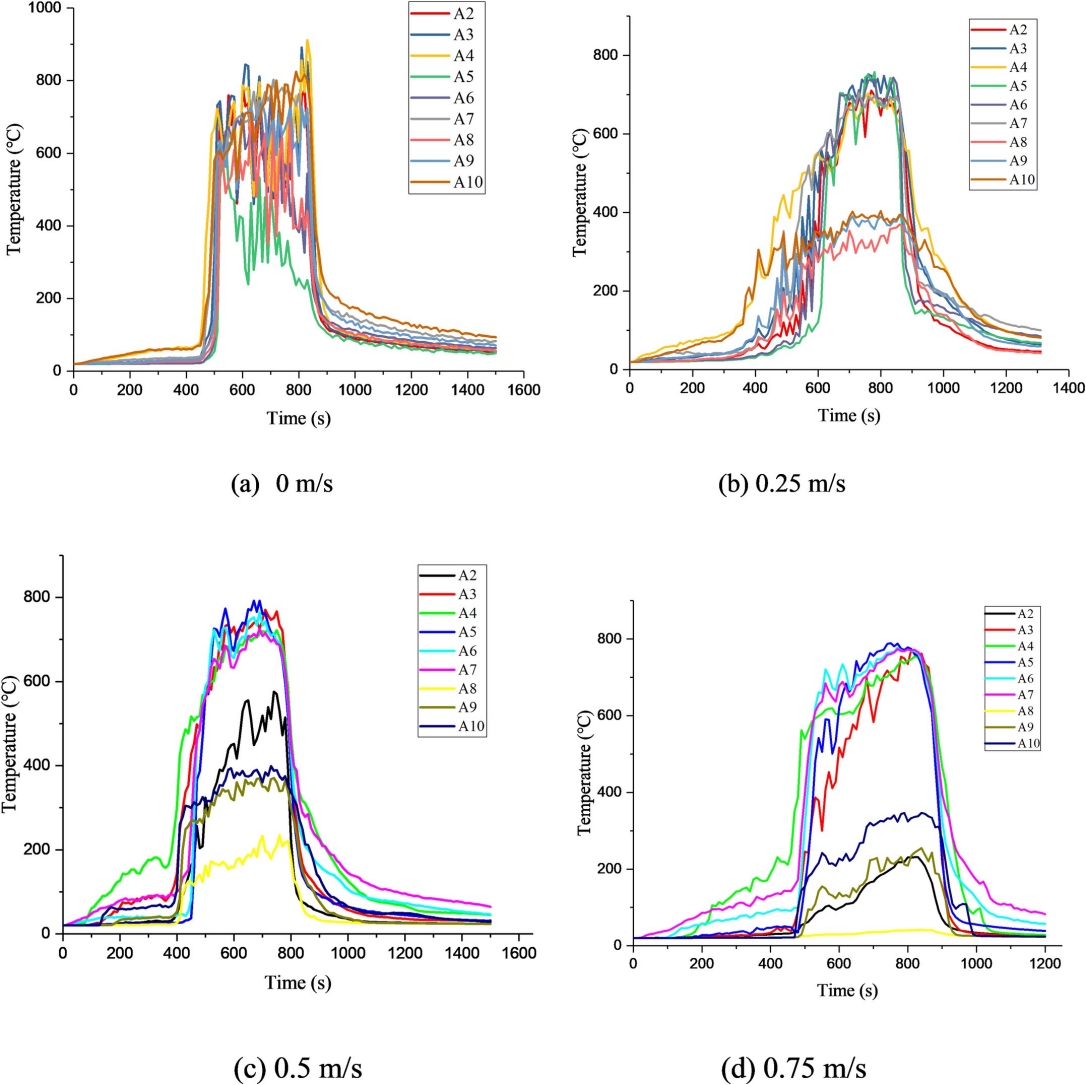
Temperature change in lining fire spreading under different wind speeds.

Fig 17 presents the horizontal smoke-temperature profiles for different wind speeds. It can be observed from Fig 17A that the temperature rises sharply between the slow-rise and stable-combustion stages under quiescent conditions because the fire is initially small and has low intensity. When heat is accumulated to a certain extent, a fire flashover occurs. Under wind speeds of 0.25 m/s, 0.5 m/s, and 0.75 m/s, sufficient oxygen for timber combustion enters the tunnel, and thus, the fire exhibits a steady growth.

**Fig 18:**
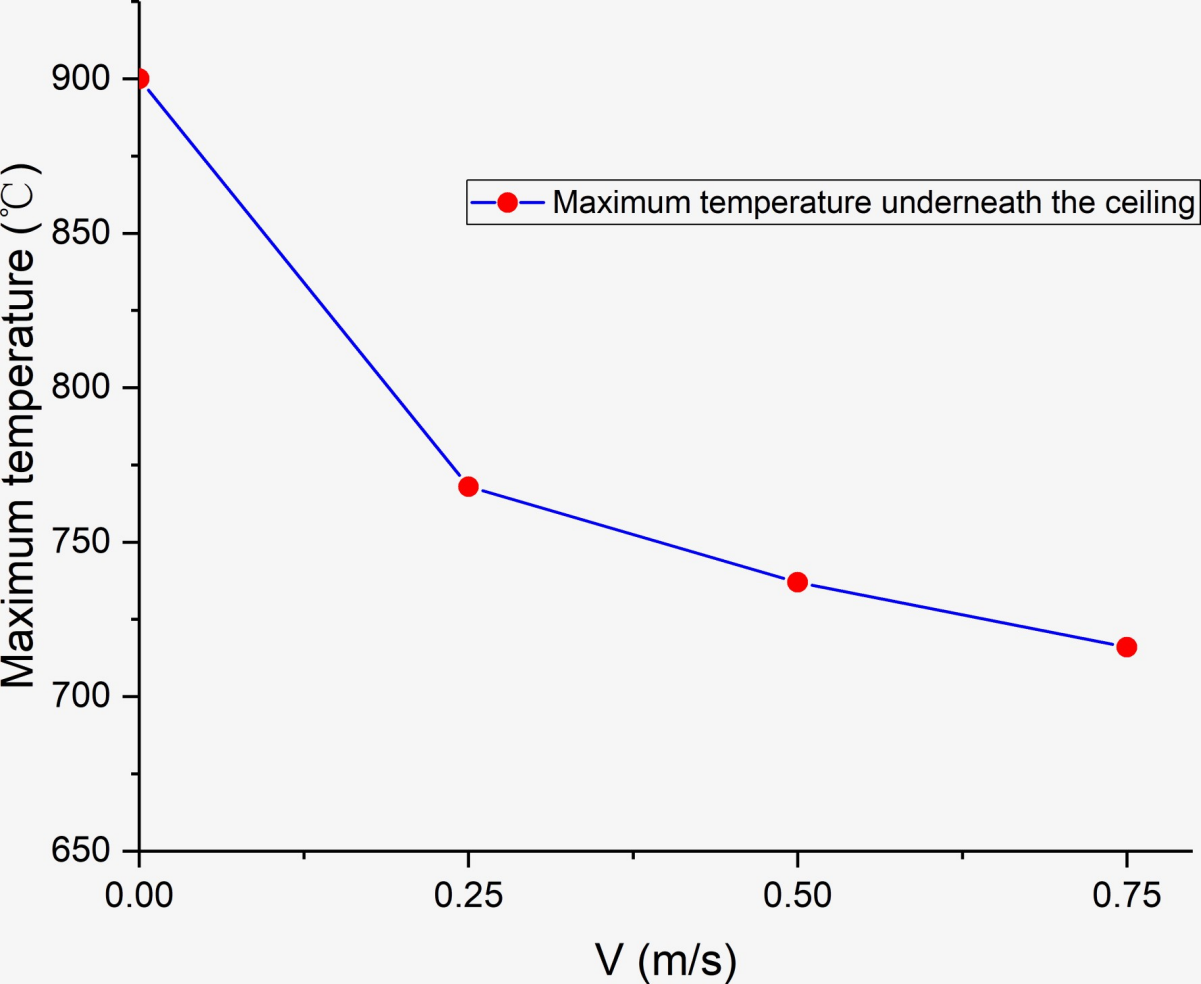
Maximum temperature underneath lining fire spreading under different wind speeds.

From Fig 18, it can be observed that the maximum smoke temperature appears underneath the timber ceilings, which can damage the tunnel structure. Under quiescent conditions, the maximum temperature near the timber ceilings is ~900°C. As the wind speed increases to 0.25 m/s, 0.5 m/s, and 0.75 m/s, the maximum temperature near the timber ceilings reaches 768, 737, and 716°C, respectively. Thus, with increase in the wind speed, the maximum temperature above the fire source decreases. This is because the accumulated high-temperature smoke is gradually dispersed with increase in the wind speed, and the wind flow dilutes the high-temperature smoke flow.

## 5. Conclusions

In this study, experimental analyses and numerical simulations of a timber-lining fire in a three-dimensional tunnel were performed, and the smoke temperature, flame characteristics, and flame-spread mechanism were investigated. The following conclusions could be drawn:

1. In the process of flame spread, the flame traverses the sidewall-ceiling-sidewall pathway in three stages along the tunnel cross-section during flame spreading. Under the influence of fire plumes, the average flame-spread rate increases along the vertical direction and decreases when the flame reaches the timber-lining corners.
2. The maximum temperature and flame lengths of the fire plume underneath the timber linings are affected by the wind speed. With increase in the wind speed, the maximum temperature above the fire source decreases. The flame length underneath the ceiling in the x-direction is longer than that in the y-direction. Moreover, as the wind speed increases, the normalized flame lengths *R*(*f*) in the two directions decrease with decrease in the wind speed.
3. The maximum temperature in the three sections is first recorded in the tunnel cross-section in the initial fire stage. Higher wind speeds correspond to the maximum-temperature points of the three sections being farther away from the fire source.

## Supporting information

S1 File The FDS program with quiescent conditions. (PDF)

S2 File The FDS program with wind speed 0.25m/s. (PDF)

S3 File The FDS program with wind speed 0.5m/s. (PDF)

S4 File The FDS program with wind speed 0.75m/s. (PDF)
