# Supplementary material for: Fire behaviors along timber linings affixed to tunnel walls in mines
Source: PLoS One. 2021 Dec 2;16(12):e0260655. doi: 10.1371/journal.pone.0260655 (PMC8639102; doi:10.1371/journal.pone.0260655)
Supplement: S1 File — (PDF) [file pone.0260655.s001.pdf]

hd01040113.fds
Generated by PyroSim - Version 2020.1.0324
2020-11-9 16:40:00

```
&HEAD CHID='hd01040113'/
&TIME T_END=1500.0/
&DUMP DT_DEVC=2.0, DT_PL3D=10.0, DT_RESTART=300.0, DT_SL3D=1.0, WRITE_XYZ=.TRUE./

&MESH ID='Mesh02', IJK=200,25,25, XB=-0.5,4.5,0.0,0.5,0.0,0.5/


&REAC ID='POLYURETHANE',
      FYI='NFPA Babrauskas',
      FUEL='REAC_FUEL',
      C=6.3,
      H=7.1,
      O=2.1,
      N=1.0,
      SOOT_YIELD=0.1/

&DEVC ID='4', QUANTITY='THERMOCOUPLE', XYZ=1.5,0.04,0.38/
&DEVC ID='3', QUANTITY='THERMOCOUPLE', XYZ=1.5,0.04,0.28/
&DEVC ID='2', QUANTITY='THERMOCOUPLE', XYZ=1.5,0.04,0.18/
&DEVC ID='401', QUANTITY='THERMOCOUPLE', XYZ=1.5,0.06,0.38/
&DEVC ID='301', QUANTITY='THERMOCOUPLE', XYZ=1.5,0.06,0.28/
&DEVC ID='201', QUANTITY='THERMOCOUPLE', XYZ=1.5,0.06,0.18/
&DEVC ID='402', QUANTITY='THERMOCOUPLE', XYZ=1.5,0.08,0.38/
&DEVC ID='302', QUANTITY='THERMOCOUPLE', XYZ=1.5,0.08,0.28/
&DEVC ID='202', QUANTITY='THERMOCOUPLE', XYZ=1.5,0.08,0.18/
&DEVC ID='403', QUANTITY='THERMOCOUPLE', XYZ=1.5,0.1,0.38/
&DEVC ID='303', QUANTITY='THERMOCOUPLE', XYZ=1.5,0.1,0.28/
&DEVC ID='203', QUANTITY='THERMOCOUPLE', XYZ=1.5,0.1,0.18/
&DEVC ID='404', QUANTITY='THERMOCOUPLE', XYZ=1.5,0.12,0.38/
&DEVC ID='304', QUANTITY='THERMOCOUPLE', XYZ=1.5,0.12,0.28/
&DEVC ID='204', QUANTITY='THERMOCOUPLE', XYZ=1.5,0.12,0.18/
&DEVC ID='405', QUANTITY='THERMOCOUPLE', XYZ=1.5,0.14,0.38/
&DEVC ID='305', QUANTITY='THERMOCOUPLE', XYZ=1.5,0.14,0.28/
&DEVC ID='205', QUANTITY='THERMOCOUPLE', XYZ=1.5,0.14,0.18/
&DEVC ID='406', QUANTITY='THERMOCOUPLE', XYZ=1.5,0.16,0.38/
&DEVC ID='306', QUANTITY='THERMOCOUPLE', XYZ=1.5,0.16,0.28/
&DEVC ID='206', QUANTITY='THERMOCOUPLE', XYZ=1.5,0.16,0.18/
&DEVC ID='407', QUANTITY='THERMOCOUPLE', XYZ=1.5,0.18,0.38/
&DEVC ID='307', QUANTITY='THERMOCOUPLE', XYZ=1.5,0.18,0.28/
```

```
&DEVC ID='207', QUANTITY='THERMOCOUPLE', XYZ=1.5,0.18,0.18/
&DEVC ID='408', QUANTITY='THERMOCOUPLE', XYZ=1.5,0.2,0.38/
&DEVC ID='308', QUANTITY='THERMOCOUPLE', XYZ=1.5,0.2,0.28/
&DEVC ID='208', QUANTITY='THERMOCOUPLE', XYZ=1.5,0.2,0.18/
&DEVC ID='409', QUANTITY='THERMOCOUPLE', XYZ=1.5,0.22,0.38/
&DEVC ID='309', QUANTITY='THERMOCOUPLE', XYZ=1.5,0.22,0.28/
&DEVC ID='209', QUANTITY='THERMOCOUPLE', XYZ=1.5,0.22,0.18/
&DEVC ID='410', QUANTITY='THERMOCOUPLE', XYZ=1.5,0.24,0.38/
&DEVC ID='310', QUANTITY='THERMOCOUPLE', XYZ=1.5,0.24,0.28/
&DEVC ID='210', QUANTITY='THERMOCOUPLE', XYZ=1.5,0.24,0.18/
&DEVC ID='411', QUANTITY='THERMOCOUPLE', XYZ=1.5,0.26,0.38/
&DEVC ID='311', QUANTITY='THERMOCOUPLE', XYZ=1.5,0.26,0.28/
&DEVC ID='211', QUANTITY='THERMOCOUPLE', XYZ=1.5,0.26,0.18/
&DEVC ID='412', QUANTITY='THERMOCOUPLE', XYZ=1.5,0.28,0.38/
&DEVC ID='312', QUANTITY='THERMOCOUPLE', XYZ=1.5,0.28,0.28/
&DEVC ID='212', QUANTITY='THERMOCOUPLE', XYZ=1.5,0.28,0.18/
&DEVC ID='413', QUANTITY='THERMOCOUPLE', XYZ=1.5,0.3,0.38/
&DEVC ID='313', QUANTITY='THERMOCOUPLE', XYZ=1.5,0.3,0.28/
&DEVC ID='213', QUANTITY='THERMOCOUPLE', XYZ=1.5,0.3,0.18/
&DEVC ID='414', QUANTITY='THERMOCOUPLE', XYZ=1.5,0.32,0.38/
&DEVC ID='314', QUANTITY='THERMOCOUPLE', XYZ=1.5,0.32,0.28/
&DEVC ID='214', QUANTITY='THERMOCOUPLE', XYZ=1.5,0.32,0.18/
&DEVC ID='415', QUANTITY='THERMOCOUPLE', XYZ=1.5,0.34,0.38/
&DEVC ID='315', QUANTITY='THERMOCOUPLE', XYZ=1.5,0.34,0.28/
&DEVC ID='215', QUANTITY='THERMOCOUPLE', XYZ=1.5,0.34,0.18/
&DEVC ID='416', QUANTITY='THERMOCOUPLE', XYZ=1.5,0.36,0.38/
&DEVC ID='316', QUANTITY='THERMOCOUPLE', XYZ=1.5,0.36,0.28/
&DEVC ID='216', QUANTITY='THERMOCOUPLE', XYZ=1.5,0.36,0.18/
&DEVC ID='417', QUANTITY='THERMOCOUPLE', XYZ=1.5,0.38,0.38/
&DEVC ID='317', QUANTITY='THERMOCOUPLE', XYZ=1.5,0.38,0.28/
&DEVC ID='217', QUANTITY='THERMOCOUPLE', XYZ=1.5,0.38,0.18/
&DEVC ID='418', QUANTITY='THERMOCOUPLE', XYZ=1.5,0.4,0.38/
&DEVC ID='318', QUANTITY='THERMOCOUPLE', XYZ=1.5,0.4,0.28/
&DEVC ID='218', QUANTITY='THERMOCOUPLE', XYZ=1.5,0.4,0.18/
&DEVC ID='419', QUANTITY='THERMOCOUPLE', XYZ=1.5,0.42,0.38/
&DEVC ID='319', QUANTITY='THERMOCOUPLE', XYZ=1.5,0.42,0.28/
&DEVC ID='219', QUANTITY='THERMOCOUPLE', XYZ=1.5,0.42,0.18/
&DEVC ID='420', QUANTITY='THERMOCOUPLE', XYZ=1.5,0.44,0.38/
&DEVC ID='320', QUANTITY='THERMOCOUPLE', XYZ=1.5,0.44,0.28/
&DEVC ID='220', QUANTITY='THERMOCOUPLE', XYZ=1.5,0.44,0.18/
&DEVC ID='421', QUANTITY='THERMOCOUPLE', XYZ=1.5,0.46,0.38/
&DEVC ID='321', QUANTITY='THERMOCOUPLE', XYZ=1.5,0.46,0.28/
&DEVC ID='221', QUANTITY='THERMOCOUPLE', XYZ=1.5,0.46,0.18/
&DEVC ID='422', QUANTITY='THERMOCOUPLE', XYZ=0.5,0.04,0.38/
```

```
&DEVC ID='322', QUANTITY='THERMOCOUPLE', XYZ=0.5,0.04,0.28/
&DEVC ID='222', QUANTITY='THERMOCOUPLE', XYZ=0.5,0.04,0.18/
&DEVC ID='40101', QUANTITY='THERMOCOUPLE', XYZ=0.5,0.06,0.38/
&DEVC ID='30101', QUANTITY='THERMOCOUPLE', XYZ=0.5,0.06,0.28/
&DEVC ID='20101', QUANTITY='THERMOCOUPLE', XYZ=0.5,0.06,0.18/
&DEVC ID='40201', QUANTITY='THERMOCOUPLE', XYZ=0.5,0.08,0.38/
&DEVC ID='30201', QUANTITY='THERMOCOUPLE', XYZ=0.5,0.08,0.28/
&DEVC ID='20201', QUANTITY='THERMOCOUPLE', XYZ=0.5,0.08,0.18/
&DEVC ID='40301', QUANTITY='THERMOCOUPLE', XYZ=0.5,0.1,0.38/
&DEVC ID='30301', QUANTITY='THERMOCOUPLE', XYZ=0.5,0.1,0.28/
&DEVC ID='20301', QUANTITY='THERMOCOUPLE', XYZ=0.5,0.1,0.18/
&DEVC ID='40401', QUANTITY='THERMOCOUPLE', XYZ=0.5,0.12,0.38/
&DEVC ID='30401', QUANTITY='THERMOCOUPLE', XYZ=0.5,0.12,0.28/
&DEVC ID='20401', QUANTITY='THERMOCOUPLE', XYZ=0.5,0.12,0.18/
&DEVC ID='40501', QUANTITY='THERMOCOUPLE', XYZ=0.5,0.14,0.38/
&DEVC ID='30501', QUANTITY='THERMOCOUPLE', XYZ=0.5,0.14,0.28/
&DEVC ID='20501', QUANTITY='THERMOCOUPLE', XYZ=0.5,0.14,0.18/
&DEVC ID='40601', QUANTITY='THERMOCOUPLE', XYZ=0.5,0.16,0.38/
&DEVC ID='30601', QUANTITY='THERMOCOUPLE', XYZ=0.5,0.16,0.28/
&DEVC ID='20601', QUANTITY='THERMOCOUPLE', XYZ=0.5,0.16,0.18/
&DEVC ID='40701', QUANTITY='THERMOCOUPLE', XYZ=0.5,0.18,0.38/
&DEVC ID='30701', QUANTITY='THERMOCOUPLE', XYZ=0.5,0.18,0.28/
&DEVC ID='20701', QUANTITY='THERMOCOUPLE', XYZ=0.5,0.18,0.18/
&DEVC ID='40801', QUANTITY='THERMOCOUPLE', XYZ=0.5,0.2,0.38/
&DEVC ID='30801', QUANTITY='THERMOCOUPLE', XYZ=0.5,0.2,0.28/
&DEVC ID='20801', QUANTITY='THERMOCOUPLE', XYZ=0.5,0.2,0.18/
&DEVC ID='40901', QUANTITY='THERMOCOUPLE', XYZ=0.5,0.22,0.38/
&DEVC ID='30901', QUANTITY='THERMOCOUPLE', XYZ=0.5,0.22,0.28/
&DEVC ID='20901', QUANTITY='THERMOCOUPLE', XYZ=0.5,0.22,0.18/
&DEVC ID='41001', QUANTITY='THERMOCOUPLE', XYZ=0.5,0.24,0.38/
&DEVC ID='31001', QUANTITY='THERMOCOUPLE', XYZ=0.5,0.24,0.28/
&DEVC ID='21001', QUANTITY='THERMOCOUPLE', XYZ=0.5,0.24,0.18/
&DEVC ID='41101', QUANTITY='THERMOCOUPLE', XYZ=0.5,0.26,0.38/
&DEVC ID='31101', QUANTITY='THERMOCOUPLE', XYZ=0.5,0.26,0.28/
&DEVC ID='21101', QUANTITY='THERMOCOUPLE', XYZ=0.5,0.26,0.18/
&DEVC ID='41201', QUANTITY='THERMOCOUPLE', XYZ=0.5,0.28,0.38/
&DEVC ID='31201', QUANTITY='THERMOCOUPLE', XYZ=0.5,0.28,0.28/
&DEVC ID='21201', QUANTITY='THERMOCOUPLE', XYZ=0.5,0.28,0.18/
&DEVC ID='41301', QUANTITY='THERMOCOUPLE', XYZ=0.5,0.3,0.38/
&DEVC ID='31301', QUANTITY='THERMOCOUPLE', XYZ=0.5,0.3,0.28/
&DEVC ID='21301', QUANTITY='THERMOCOUPLE', XYZ=0.5,0.3,0.18/
&DEVC ID='41401', QUANTITY='THERMOCOUPLE', XYZ=0.5,0.32,0.38/
&DEVC ID='31401', QUANTITY='THERMOCOUPLE', XYZ=0.5,0.32,0.28/
&DEVC ID='21401', QUANTITY='THERMOCOUPLE', XYZ=0.5,0.32,0.18/
```

&DEVC ID='41501', QUANTITY='THERMOCOUPLE', XYZ=0.5,0.34,0.38/
&DEVC ID='31501', QUANTITY='THERMOCOUPLE', XYZ=0.5,0.34,0.28/
&DEVC ID='21501', QUANTITY='THERMOCOUPLE', XYZ=0.5,0.34,0.18/
&DEVC ID='41601', QUANTITY='THERMOCOUPLE', XYZ=0.5,0.36,0.38/
&DEVC ID='31601', QUANTITY='THERMOCOUPLE', XYZ=0.5,0.36,0.28/
&DEVC ID='21601', QUANTITY='THERMOCOUPLE', XYZ=0.5,0.36,0.18/
&DEVC ID='41701', QUANTITY='THERMOCOUPLE', XYZ=0.5,0.38,0.38/
&DEVC ID='31701', QUANTITY='THERMOCOUPLE', XYZ=0.5,0.38,0.28/
&DEVC ID='21701', QUANTITY='THERMOCOUPLE', XYZ=0.5,0.38,0.18/
&DEVC ID='41801', QUANTITY='THERMOCOUPLE', XYZ=0.5,0.4,0.38/
&DEVC ID='31801', QUANTITY='THERMOCOUPLE', XYZ=0.5,0.4,0.28/
&DEVC ID='21801', QUANTITY='THERMOCOUPLE', XYZ=0.5,0.4,0.18/
&DEVC ID='41901', QUANTITY='THERMOCOUPLE', XYZ=0.5,0.42,0.38/
&DEVC ID='31901', QUANTITY='THERMOCOUPLE', XYZ=0.5,0.42,0.28/
&DEVC ID='21901', QUANTITY='THERMOCOUPLE', XYZ=0.5,0.42,0.18/
&DEVC ID='42001', QUANTITY='THERMOCOUPLE', XYZ=0.5,0.44,0.38/
&DEVC ID='32001', QUANTITY='THERMOCOUPLE', XYZ=0.5,0.44,0.28/
&DEVC ID='22001', QUANTITY='THERMOCOUPLE', XYZ=0.5,0.44,0.18/
&DEVC ID='42101', QUANTITY='THERMOCOUPLE', XYZ=0.5,0.46,0.38/
&DEVC ID='32101', QUANTITY='THERMOCOUPLE', XYZ=0.5,0.46,0.28/
&DEVC ID='22101', QUANTITY='THERMOCOUPLE', XYZ=0.5,0.46,0.18/
&DEVC ID='423', QUANTITY='THERMOCOUPLE', XYZ=2.5,0.04,0.38/
&DEVC ID='323', QUANTITY='THERMOCOUPLE', XYZ=2.5,0.04,0.28/
&DEVC ID='223', QUANTITY='THERMOCOUPLE', XYZ=2.5,0.04,0.18/
&DEVC ID='40102', QUANTITY='THERMOCOUPLE', XYZ=2.5,0.06,0.38/
&DEVC ID='30102', QUANTITY='THERMOCOUPLE', XYZ=2.5,0.06,0.28/
&DEVC ID='20102', QUANTITY='THERMOCOUPLE', XYZ=2.5,0.06,0.18/
&DEVC ID='40202', QUANTITY='THERMOCOUPLE', XYZ=2.5,0.08,0.38/
&DEVC ID='30202', QUANTITY='THERMOCOUPLE', XYZ=2.5,0.08,0.28/
&DEVC ID='20202', QUANTITY='THERMOCOUPLE', XYZ=2.5,0.08,0.18/
&DEVC ID='40302', QUANTITY='THERMOCOUPLE', XYZ=2.5,0.1,0.38/
&DEVC ID='30302', QUANTITY='THERMOCOUPLE', XYZ=2.5,0.1,0.28/
&DEVC ID='20302', QUANTITY='THERMOCOUPLE', XYZ=2.5,0.1,0.18/
&DEVC ID='40402', QUANTITY='THERMOCOUPLE', XYZ=2.5,0.12,0.38/
&DEVC ID='30402', QUANTITY='THERMOCOUPLE', XYZ=2.5,0.12,0.28/
&DEVC ID='20402', QUANTITY='THERMOCOUPLE', XYZ=2.5,0.12,0.18/
&DEVC ID='40502', QUANTITY='THERMOCOUPLE', XYZ=2.5,0.14,0.38/
&DEVC ID='30502', QUANTITY='THERMOCOUPLE', XYZ=2.5,0.14,0.28/
&DEVC ID='20502', QUANTITY='THERMOCOUPLE', XYZ=2.5,0.14,0.18/
&DEVC ID='40602', QUANTITY='THERMOCOUPLE', XYZ=2.5,0.16,0.38/
&DEVC ID='30602', QUANTITY='THERMOCOUPLE', XYZ=2.5,0.16,0.28/
&DEVC ID='20602', QUANTITY='THERMOCOUPLE', XYZ=2.5,0.16,0.18/
&DEVC ID='40702', QUANTITY='THERMOCOUPLE', XYZ=2.5,0.18,0.38/
&DEVC ID='30702', QUANTITY='THERMOCOUPLE', XYZ=2.5,0.18,0.28/

```
&DEVC ID='20702', QUANTITY='THERMOCOUPLE', XYZ=2.5,0.18,0.18/
&DEVC ID='40802', QUANTITY='THERMOCOUPLE', XYZ=2.5,0.2,0.38/
&DEVC ID='30802', QUANTITY='THERMOCOUPLE', XYZ=2.5,0.2,0.28/
&DEVC ID='20802', QUANTITY='THERMOCOUPLE', XYZ=2.5,0.2,0.18/
&DEVC ID='40902', QUANTITY='THERMOCOUPLE', XYZ=2.5,0.22,0.38/
&DEVC ID='30902', QUANTITY='THERMOCOUPLE', XYZ=2.5,0.22,0.28/
&DEVC ID='20902', QUANTITY='THERMOCOUPLE', XYZ=2.5,0.22,0.18/
&DEVC ID='41002', QUANTITY='THERMOCOUPLE', XYZ=2.5,0.24,0.38/
&DEVC ID='31002', QUANTITY='THERMOCOUPLE', XYZ=2.5,0.24,0.28/
&DEVC ID='21002', QUANTITY='THERMOCOUPLE', XYZ=2.5,0.24,0.18/
&DEVC ID='41102', QUANTITY='THERMOCOUPLE', XYZ=2.5,0.26,0.38/
&DEVC ID='31102', QUANTITY='THERMOCOUPLE', XYZ=2.5,0.26,0.28/
&DEVC ID='21102', QUANTITY='THERMOCOUPLE', XYZ=2.5,0.26,0.18/
&DEVC ID='41202', QUANTITY='THERMOCOUPLE', XYZ=2.5,0.28,0.38/
&DEVC ID='31202', QUANTITY='THERMOCOUPLE', XYZ=2.5,0.28,0.28/
&DEVC ID='21202', QUANTITY='THERMOCOUPLE', XYZ=2.5,0.28,0.18/
&DEVC ID='41302', QUANTITY='THERMOCOUPLE', XYZ=2.5,0.3,0.38/
&DEVC ID='31302', QUANTITY='THERMOCOUPLE', XYZ=2.5,0.3,0.28/
&DEVC ID='21302', QUANTITY='THERMOCOUPLE', XYZ=2.5,0.3,0.18/
&DEVC ID='41402', QUANTITY='THERMOCOUPLE', XYZ=2.5,0.32,0.38/
&DEVC ID='31402', QUANTITY='THERMOCOUPLE', XYZ=2.5,0.32,0.28/
&DEVC ID='21402', QUANTITY='THERMOCOUPLE', XYZ=2.5,0.32,0.18/
&DEVC ID='41502', QUANTITY='THERMOCOUPLE', XYZ=2.5,0.34,0.38/
&DEVC ID='31502', QUANTITY='THERMOCOUPLE', XYZ=2.5,0.34,0.28/
&DEVC ID='21502', QUANTITY='THERMOCOUPLE', XYZ=2.5,0.34,0.18/
&DEVC ID='41602', QUANTITY='THERMOCOUPLE', XYZ=2.5,0.36,0.38/
&DEVC ID='31602', QUANTITY='THERMOCOUPLE', XYZ=2.5,0.36,0.28/
&DEVC ID='21602', QUANTITY='THERMOCOUPLE', XYZ=2.5,0.36,0.18/
&DEVC ID='41702', QUANTITY='THERMOCOUPLE', XYZ=2.5,0.38,0.38/
&DEVC ID='31702', QUANTITY='THERMOCOUPLE', XYZ=2.5,0.38,0.28/
&DEVC ID='21702', QUANTITY='THERMOCOUPLE', XYZ=2.5,0.38,0.18/
&DEVC ID='41802', QUANTITY='THERMOCOUPLE', XYZ=2.5,0.4,0.38/
&DEVC ID='31802', QUANTITY='THERMOCOUPLE', XYZ=2.5,0.4,0.28/
&DEVC ID='21802', QUANTITY='THERMOCOUPLE', XYZ=2.5,0.4,0.18/
&DEVC ID='41902', QUANTITY='THERMOCOUPLE', XYZ=2.5,0.42,0.38/
&DEVC ID='31902', QUANTITY='THERMOCOUPLE', XYZ=2.5,0.42,0.28/
&DEVC ID='21902', QUANTITY='THERMOCOUPLE', XYZ=2.5,0.42,0.18/
&DEVC ID='42002', QUANTITY='THERMOCOUPLE', XYZ=2.5,0.44,0.38/
&DEVC ID='32002', QUANTITY='THERMOCOUPLE', XYZ=2.5,0.44,0.28/
&DEVC ID='22002', QUANTITY='THERMOCOUPLE', XYZ=2.5,0.44,0.18/
&DEVC ID='42102', QUANTITY='THERMOCOUPLE', XYZ=2.5,0.46,0.38/
&DEVC ID='32102', QUANTITY='THERMOCOUPLE', XYZ=2.5,0.46,0.28/
&DEVC ID='22102', QUANTITY='THERMOCOUPLE', XYZ=2.5,0.46,0.18/
&DEVC ID='THCP390', QUANTITY='THERMOCOUPLE', XYZ=1.95,0.47,0.4/
```

```
&DEVC ID='THCP211', QUANTITY='THERMOCOUPLE', XYZ=1.0,0.47,0.0/
&DEVC ID='THCP212', QUANTITY='THERMOCOUPLE', XYZ=1.05,0.47,0.0/
&DEVC ID='THCP213', QUANTITY='THERMOCOUPLE', XYZ=1.1,0.47,0.0/
&DEVC ID='THCP214', QUANTITY='THERMOCOUPLE', XYZ=1.15,0.47,0.0/
&DEVC ID='THCP215', QUANTITY='THERMOCOUPLE', XYZ=1.2,0.47,0.0/
&DEVC ID='THCP216', QUANTITY='THERMOCOUPLE', XYZ=1.25,0.47,0.0/
&DEVC ID='THCP217', QUANTITY='THERMOCOUPLE', XYZ=1.3,0.47,0.0/
&DEVC ID='THCP218', QUANTITY='THERMOCOUPLE', XYZ=1.35,0.47,0.0/
&DEVC ID='THCP219', QUANTITY='THERMOCOUPLE', XYZ=1.4,0.47,0.0/
&DEVC ID='THCP220', QUANTITY='THERMOCOUPLE', XYZ=1.45,0.47,0.0/
&DEVC ID='THCP221', QUANTITY='THERMOCOUPLE', XYZ=1.5,0.47,0.0/
&DEVC ID='THCP222', QUANTITY='THERMOCOUPLE', XYZ=1.55,0.47,0.0/
&DEVC ID='THCP223', QUANTITY='THERMOCOUPLE', XYZ=1.6,0.47,0.0/
&DEVC ID='THCP224', QUANTITY='THERMOCOUPLE', XYZ=1.65,0.47,0.0/
&DEVC ID='THCP225', QUANTITY='THERMOCOUPLE', XYZ=1.7,0.47,0.0/
&DEVC ID='THCP226', QUANTITY='THERMOCOUPLE', XYZ=1.75,0.47,0.0/
&DEVC ID='THCP227', QUANTITY='THERMOCOUPLE', XYZ=1.8,0.47,0.0/
&DEVC ID='THCP228', QUANTITY='THERMOCOUPLE', XYZ=1.85,0.47,0.0/
&DEVC ID='THCP229', QUANTITY='THERMOCOUPLE', XYZ=1.9,0.47,0.0/
&DEVC ID='THCP230', QUANTITY='THERMOCOUPLE', XYZ=1.95,0.47,0.0/
&DEVC ID='THCP231', QUANTITY='THERMOCOUPLE', XYZ=1.0,0.47,0.05/
&DEVC ID='THCP232', QUANTITY='THERMOCOUPLE', XYZ=1.05,0.47,0.05/
&DEVC ID='THCP233', QUANTITY='THERMOCOUPLE', XYZ=1.1,0.47,0.05/
&DEVC ID='THCP234', QUANTITY='THERMOCOUPLE', XYZ=1.15,0.47,0.05/
&DEVC ID='THCP235', QUANTITY='THERMOCOUPLE', XYZ=1.2,0.47,0.05/
&DEVC ID='THCP236', QUANTITY='THERMOCOUPLE', XYZ=1.25,0.47,0.05/
&DEVC ID='THCP237', QUANTITY='THERMOCOUPLE', XYZ=1.3,0.47,0.05/
&DEVC ID='THCP238', QUANTITY='THERMOCOUPLE', XYZ=1.35,0.47,0.05/
&DEVC ID='THCP239', QUANTITY='THERMOCOUPLE', XYZ=1.4,0.47,0.05/
&DEVC ID='THCP240', QUANTITY='THERMOCOUPLE', XYZ=1.45,0.47,0.05/
&DEVC ID='THCP241', QUANTITY='THERMOCOUPLE', XYZ=1.5,0.47,0.05/
&DEVC ID='THCP242', QUANTITY='THERMOCOUPLE', XYZ=1.55,0.47,0.05/
&DEVC ID='THCP243', QUANTITY='THERMOCOUPLE', XYZ=1.6,0.47,0.05/
&DEVC ID='THCP244', QUANTITY='THERMOCOUPLE', XYZ=1.65,0.47,0.05/
&DEVC ID='THCP245', QUANTITY='THERMOCOUPLE', XYZ=1.7,0.47,0.05/
&DEVC ID='THCP246', QUANTITY='THERMOCOUPLE', XYZ=1.75,0.47,0.05/
&DEVC ID='THCP247', QUANTITY='THERMOCOUPLE', XYZ=1.8,0.47,0.05/
&DEVC ID='THCP248', QUANTITY='THERMOCOUPLE', XYZ=1.85,0.47,0.05/
&DEVC ID='THCP249', QUANTITY='THERMOCOUPLE', XYZ=1.9,0.47,0.05/
&DEVC ID='THCP250', QUANTITY='THERMOCOUPLE', XYZ=1.95,0.47,0.05/
&DEVC ID='THCP251', QUANTITY='THERMOCOUPLE', XYZ=1.0,0.47,0.1/
&DEVC ID='THCP252', QUANTITY='THERMOCOUPLE', XYZ=1.05,0.47,0.1/
&DEVC ID='THCP253', QUANTITY='THERMOCOUPLE', XYZ=1.1,0.47,0.1/
&DEVC ID='THCP254', QUANTITY='THERMOCOUPLE', XYZ=1.15,0.47,0.1/
```

```
&DEVC ID='THCP255', QUANTITY='THERMOCOUPLE', XYZ=1.2,0.47,0.1/
&DEVC ID='THCP256', QUANTITY='THERMOCOUPLE', XYZ=1.25,0.47,0.1/
&DEVC ID='THCP257', QUANTITY='THERMOCOUPLE', XYZ=1.3,0.47,0.1/
&DEVC ID='THCP258', QUANTITY='THERMOCOUPLE', XYZ=1.35,0.47,0.1/
&DEVC ID='THCP259', QUANTITY='THERMOCOUPLE', XYZ=1.4,0.47,0.1/
&DEVC ID='THCP260', QUANTITY='THERMOCOUPLE', XYZ=1.45,0.47,0.1/
&DEVC ID='THCP261', QUANTITY='THERMOCOUPLE', XYZ=1.5,0.47,0.1/
&DEVC ID='THCP262', QUANTITY='THERMOCOUPLE', XYZ=1.55,0.47,0.1/
&DEVC ID='THCP263', QUANTITY='THERMOCOUPLE', XYZ=1.6,0.47,0.1/
&DEVC ID='THCP264', QUANTITY='THERMOCOUPLE', XYZ=1.65,0.47,0.1/
&DEVC ID='THCP265', QUANTITY='THERMOCOUPLE', XYZ=1.7,0.47,0.1/
&DEVC ID='THCP266', QUANTITY='THERMOCOUPLE', XYZ=1.75,0.47,0.1/
&DEVC ID='THCP267', QUANTITY='THERMOCOUPLE', XYZ=1.8,0.47,0.1/
&DEVC ID='THCP268', QUANTITY='THERMOCOUPLE', XYZ=1.85,0.47,0.1/
&DEVC ID='THCP269', QUANTITY='THERMOCOUPLE', XYZ=1.9,0.47,0.1/
&DEVC ID='THCP270', QUANTITY='THERMOCOUPLE', XYZ=1.95,0.47,0.1/
&DEVC ID='THCP271', QUANTITY='THERMOCOUPLE', XYZ=1.0,0.47,0.15/
&DEVC ID='THCP272', QUANTITY='THERMOCOUPLE', XYZ=1.05,0.47,0.15/
&DEVC ID='THCP273', QUANTITY='THERMOCOUPLE', XYZ=1.1,0.47,0.15/
&DEVC ID='THCP274', QUANTITY='THERMOCOUPLE', XYZ=1.15,0.47,0.15/
&DEVC ID='THCP275', QUANTITY='THERMOCOUPLE', XYZ=1.2,0.47,0.15/
&DEVC ID='THCP276', QUANTITY='THERMOCOUPLE', XYZ=1.25,0.47,0.15/
&DEVC ID='THCP277', QUANTITY='THERMOCOUPLE', XYZ=1.3,0.47,0.15/
&DEVC ID='THCP278', QUANTITY='THERMOCOUPLE', XYZ=1.35,0.47,0.15/
&DEVC ID='THCP279', QUANTITY='THERMOCOUPLE', XYZ=1.4,0.47,0.15/
&DEVC ID='THCP280', QUANTITY='THERMOCOUPLE', XYZ=1.45,0.47,0.15/
&DEVC ID='THCP281', QUANTITY='THERMOCOUPLE', XYZ=1.5,0.47,0.15/
&DEVC ID='THCP282', QUANTITY='THERMOCOUPLE', XYZ=1.55,0.47,0.15/
&DEVC ID='THCP283', QUANTITY='THERMOCOUPLE', XYZ=1.6,0.47,0.15/
&DEVC ID='THCP284', QUANTITY='THERMOCOUPLE', XYZ=1.65,0.47,0.15/
&DEVC ID='THCP285', QUANTITY='THERMOCOUPLE', XYZ=1.7,0.47,0.15/
&DEVC ID='THCP286', QUANTITY='THERMOCOUPLE', XYZ=1.75,0.47,0.15/
&DEVC ID='THCP287', QUANTITY='THERMOCOUPLE', XYZ=1.8,0.47,0.15/
&DEVC ID='THCP288', QUANTITY='THERMOCOUPLE', XYZ=1.85,0.47,0.15/
&DEVC ID='THCP289', QUANTITY='THERMOCOUPLE', XYZ=1.9,0.47,0.15/
&DEVC ID='THCP290', QUANTITY='THERMOCOUPLE', XYZ=1.95,0.47,0.15/
&DEVC ID='THCP291', QUANTITY='THERMOCOUPLE', XYZ=1.0,0.47,0.2/
&DEVC ID='THCP292', QUANTITY='THERMOCOUPLE', XYZ=1.05,0.47,0.2/
&DEVC ID='THCP293', QUANTITY='THERMOCOUPLE', XYZ=1.1,0.47,0.2/
&DEVC ID='THCP294', QUANTITY='THERMOCOUPLE', XYZ=1.15,0.47,0.2/
&DEVC ID='THCP295', QUANTITY='THERMOCOUPLE', XYZ=1.2,0.47,0.2/
&DEVC ID='THCP296', QUANTITY='THERMOCOUPLE', XYZ=1.25,0.47,0.2/
&DEVC ID='THCP297', QUANTITY='THERMOCOUPLE', XYZ=1.3,0.47,0.2/
&DEVC ID='THCP298', QUANTITY='THERMOCOUPLE', XYZ=1.35,0.47,0.2/
```

```
&DEVC ID='THCP299', QUANTITY='THERMOCOUPLE', XYZ=1.4,0.47,0.2/
&DEVC ID='THCP300', QUANTITY='THERMOCOUPLE', XYZ=1.45,0.47,0.2/
&DEVC ID='THCP301', QUANTITY='THERMOCOUPLE', XYZ=1.5,0.47,0.2/
&DEVC ID='THCP302', QUANTITY='THERMOCOUPLE', XYZ=1.55,0.47,0.2/
&DEVC ID='THCP303', QUANTITY='THERMOCOUPLE', XYZ=1.6,0.47,0.2/
&DEVC ID='THCP304', QUANTITY='THERMOCOUPLE', XYZ=1.65,0.47,0.2/
&DEVC ID='THCP305', QUANTITY='THERMOCOUPLE', XYZ=1.7,0.47,0.2/
&DEVC ID='THCP306', QUANTITY='THERMOCOUPLE', XYZ=1.75,0.47,0.2/
&DEVC ID='THCP307', QUANTITY='THERMOCOUPLE', XYZ=1.8,0.47,0.2/
&DEVC ID='THCP308', QUANTITY='THERMOCOUPLE', XYZ=1.85,0.47,0.2/
&DEVC ID='THCP309', QUANTITY='THERMOCOUPLE', XYZ=1.9,0.47,0.2/
&DEVC ID='THCP310', QUANTITY='THERMOCOUPLE', XYZ=1.95,0.47,0.2/
&DEVC ID='THCP311', QUANTITY='THERMOCOUPLE', XYZ=1.0,0.47,0.25/
&DEVC ID='THCP312', QUANTITY='THERMOCOUPLE', XYZ=1.05,0.47,0.25/
&DEVC ID='THCP313', QUANTITY='THERMOCOUPLE', XYZ=1.1,0.47,0.25/
&DEVC ID='THCP314', QUANTITY='THERMOCOUPLE', XYZ=1.15,0.47,0.25/
&DEVC ID='THCP315', QUANTITY='THERMOCOUPLE', XYZ=1.2,0.47,0.25/
&DEVC ID='THCP316', QUANTITY='THERMOCOUPLE', XYZ=1.25,0.47,0.25/
&DEVC ID='THCP317', QUANTITY='THERMOCOUPLE', XYZ=1.3,0.47,0.25/
&DEVC ID='THCP318', QUANTITY='THERMOCOUPLE', XYZ=1.35,0.47,0.25/
&DEVC ID='THCP319', QUANTITY='THERMOCOUPLE', XYZ=1.4,0.47,0.25/
&DEVC ID='THCP320', QUANTITY='THERMOCOUPLE', XYZ=1.45,0.47,0.25/
&DEVC ID='THCP321', QUANTITY='THERMOCOUPLE', XYZ=1.5,0.47,0.25/
&DEVC ID='THCP322', QUANTITY='THERMOCOUPLE', XYZ=1.55,0.47,0.25/
&DEVC ID='THCP323', QUANTITY='THERMOCOUPLE', XYZ=1.6,0.47,0.25/
&DEVC ID='THCP324', QUANTITY='THERMOCOUPLE', XYZ=1.65,0.47,0.25/
&DEVC ID='THCP325', QUANTITY='THERMOCOUPLE', XYZ=1.7,0.47,0.25/
&DEVC ID='THCP326', QUANTITY='THERMOCOUPLE', XYZ=1.75,0.47,0.25/
&DEVC ID='THCP327', QUANTITY='THERMOCOUPLE', XYZ=1.8,0.47,0.25/
&DEVC ID='THCP328', QUANTITY='THERMOCOUPLE', XYZ=1.85,0.47,0.25/
&DEVC ID='THCP329', QUANTITY='THERMOCOUPLE', XYZ=1.9,0.47,0.25/
&DEVC ID='THCP330', QUANTITY='THERMOCOUPLE', XYZ=1.95,0.47,0.25/
&DEVC ID='THCP331', QUANTITY='THERMOCOUPLE', XYZ=1.0,0.47,0.3/
&DEVC ID='THCP332', QUANTITY='THERMOCOUPLE', XYZ=1.05,0.47,0.3/
&DEVC ID='THCP333', QUANTITY='THERMOCOUPLE', XYZ=1.1,0.47,0.3/
&DEVC ID='THCP334', QUANTITY='THERMOCOUPLE', XYZ=1.15,0.47,0.3/
&DEVC ID='THCP335', QUANTITY='THERMOCOUPLE', XYZ=1.2,0.47,0.3/
&DEVC ID='THCP336', QUANTITY='THERMOCOUPLE', XYZ=1.25,0.47,0.3/
&DEVC ID='THCP337', QUANTITY='THERMOCOUPLE', XYZ=1.3,0.47,0.3/
&DEVC ID='THCP338', QUANTITY='THERMOCOUPLE', XYZ=1.35,0.47,0.3/
&DEVC ID='THCP339', QUANTITY='THERMOCOUPLE', XYZ=1.4,0.47,0.3/
&DEVC ID='THCP340', QUANTITY='THERMOCOUPLE', XYZ=1.45,0.47,0.3/
&DEVC ID='THCP341', QUANTITY='THERMOCOUPLE', XYZ=1.5,0.47,0.3/
&DEVC ID='THCP342', QUANTITY='THERMOCOUPLE', XYZ=1.55,0.47,0.3/
```

```
&DEVC ID='THCP343', QUANTITY='THERMOCOUPLE', XYZ=1.6,0.47,0.3/
&DEVC ID='THCP344', QUANTITY='THERMOCOUPLE', XYZ=1.65,0.47,0.3/
&DEVC ID='THCP345', QUANTITY='THERMOCOUPLE', XYZ=1.7,0.47,0.3/
&DEVC ID='THCP346', QUANTITY='THERMOCOUPLE', XYZ=1.75,0.47,0.3/
&DEVC ID='THCP347', QUANTITY='THERMOCOUPLE', XYZ=1.8,0.47,0.3/
&DEVC ID='THCP348', QUANTITY='THERMOCOUPLE', XYZ=1.85,0.47,0.3/
&DEVC ID='THCP349', QUANTITY='THERMOCOUPLE', XYZ=1.9,0.47,0.3/
&DEVC ID='THCP350', QUANTITY='THERMOCOUPLE', XYZ=1.95,0.47,0.3/
&DEVC ID='THCP351', QUANTITY='THERMOCOUPLE', XYZ=1.0,0.47,0.35/
&DEVC ID='THCP352', QUANTITY='THERMOCOUPLE', XYZ=1.05,0.47,0.35/
&DEVC ID='THCP353', QUANTITY='THERMOCOUPLE', XYZ=1.1,0.47,0.35/
&DEVC ID='THCP354', QUANTITY='THERMOCOUPLE', XYZ=1.15,0.47,0.35/
&DEVC ID='THCP355', QUANTITY='THERMOCOUPLE', XYZ=1.2,0.47,0.35/
&DEVC ID='THCP356', QUANTITY='THERMOCOUPLE', XYZ=1.25,0.47,0.35/
&DEVC ID='THCP357', QUANTITY='THERMOCOUPLE', XYZ=1.3,0.47,0.35/
&DEVC ID='THCP358', QUANTITY='THERMOCOUPLE', XYZ=1.35,0.47,0.35/
&DEVC ID='THCP359', QUANTITY='THERMOCOUPLE', XYZ=1.4,0.47,0.35/
&DEVC ID='THCP360', QUANTITY='THERMOCOUPLE', XYZ=1.45,0.47,0.35/
&DEVC ID='THCP361', QUANTITY='THERMOCOUPLE', XYZ=1.5,0.47,0.35/
&DEVC ID='THCP362', QUANTITY='THERMOCOUPLE', XYZ=1.55,0.47,0.35/
&DEVC ID='THCP363', QUANTITY='THERMOCOUPLE', XYZ=1.6,0.47,0.35/
&DEVC ID='THCP364', QUANTITY='THERMOCOUPLE', XYZ=1.65,0.47,0.35/
&DEVC ID='THCP365', QUANTITY='THERMOCOUPLE', XYZ=1.7,0.47,0.35/
&DEVC ID='THCP366', QUANTITY='THERMOCOUPLE', XYZ=1.75,0.47,0.35/
&DEVC ID='THCP367', QUANTITY='THERMOCOUPLE', XYZ=1.8,0.47,0.35/
&DEVC ID='THCP368', QUANTITY='THERMOCOUPLE', XYZ=1.85,0.47,0.35/
&DEVC ID='THCP369', QUANTITY='THERMOCOUPLE', XYZ=1.9,0.47,0.35/
&DEVC ID='THCP370', QUANTITY='THERMOCOUPLE', XYZ=1.95,0.47,0.35/
&DEVC ID='THCP371', QUANTITY='THERMOCOUPLE', XYZ=1.0,0.47,0.4/
&DEVC ID='THCP372', QUANTITY='THERMOCOUPLE', XYZ=1.05,0.47,0.4/
&DEVC ID='THCP373', QUANTITY='THERMOCOUPLE', XYZ=1.1,0.47,0.4/
&DEVC ID='THCP374', QUANTITY='THERMOCOUPLE', XYZ=1.15,0.47,0.4/
&DEVC ID='THCP375', QUANTITY='THERMOCOUPLE', XYZ=1.2,0.47,0.4/
&DEVC ID='THCP376', QUANTITY='THERMOCOUPLE', XYZ=1.25,0.47,0.4/
&DEVC ID='THCP377', QUANTITY='THERMOCOUPLE', XYZ=1.3,0.47,0.4/
&DEVC ID='THCP378', QUANTITY='THERMOCOUPLE', XYZ=1.35,0.47,0.4/
&DEVC ID='THCP379', QUANTITY='THERMOCOUPLE', XYZ=1.4,0.47,0.4/
&DEVC ID='THCP380', QUANTITY='THERMOCOUPLE', XYZ=1.45,0.47,0.4/
&DEVC ID='THCP381', QUANTITY='THERMOCOUPLE', XYZ=1.5,0.47,0.4/
&DEVC ID='THCP382', QUANTITY='THERMOCOUPLE', XYZ=1.55,0.47,0.4/
&DEVC ID='THCP383', QUANTITY='THERMOCOUPLE', XYZ=1.6,0.47,0.4/
&DEVC ID='THCP384', QUANTITY='THERMOCOUPLE', XYZ=1.65,0.47,0.4/
&DEVC ID='THCP385', QUANTITY='THERMOCOUPLE', XYZ=1.7,0.47,0.4/
&DEVC ID='THCP386', QUANTITY='THERMOCOUPLE', XYZ=1.75,0.47,0.4/
```

&DEVC ID='THCP387', QUANTITY='THERMOCOUPLE', XYZ=1.8,0.47,0.4/
&DEVC ID='THCP388', QUANTITY='THERMOCOUPLE', XYZ=1.85,0.47,0.4/
&DEVC ID='THCP389', QUANTITY='THERMOCOUPLE', XYZ=1.9,0.47,0.4/
&DEVC ID='THCP530', QUANTITY='THERMOCOUPLE', XYZ=1.95,0.03,0.47/
&DEVC ID='THCP411', QUANTITY='THERMOCOUPLE', XYZ=1.0,0.47,0.47/
&DEVC ID='THCP412', QUANTITY='THERMOCOUPLE', XYZ=1.05,0.47,0.47/
&DEVC ID='THCP413', QUANTITY='THERMOCOUPLE', XYZ=1.1,0.47,0.47/
&DEVC ID='THCP414', QUANTITY='THERMOCOUPLE', XYZ=1.15,0.47,0.47/
&DEVC ID='THCP415', QUANTITY='THERMOCOUPLE', XYZ=1.2,0.47,0.47/
&DEVC ID='THCP416', QUANTITY='THERMOCOUPLE', XYZ=1.25,0.47,0.47/
&DEVC ID='THCP417', QUANTITY='THERMOCOUPLE', XYZ=1.3,0.47,0.47/
&DEVC ID='THCP418', QUANTITY='THERMOCOUPLE', XYZ=1.35,0.47,0.47/
&DEVC ID='THCP419', QUANTITY='THERMOCOUPLE', XYZ=1.4,0.47,0.47/
&DEVC ID='THCP420', QUANTITY='THERMOCOUPLE', XYZ=1.45,0.47,0.47/
&DEVC ID='THCP421', QUANTITY='THERMOCOUPLE', XYZ=1.5,0.47,0.47/
&DEVC ID='THCP422', QUANTITY='THERMOCOUPLE', XYZ=1.55,0.47,0.47/
&DEVC ID='THCP423', QUANTITY='THERMOCOUPLE', XYZ=1.6,0.47,0.47/
&DEVC ID='THCP424', QUANTITY='THERMOCOUPLE', XYZ=1.65,0.47,0.47/
&DEVC ID='THCP425', QUANTITY='THERMOCOUPLE', XYZ=1.7,0.47,0.47/
&DEVC ID='THCP426', QUANTITY='THERMOCOUPLE', XYZ=1.75,0.47,0.47/
&DEVC ID='THCP427', QUANTITY='THERMOCOUPLE', XYZ=1.8,0.47,0.47/
&DEVC ID='THCP428', QUANTITY='THERMOCOUPLE', XYZ=1.85,0.47,0.47/
&DEVC ID='THCP429', QUANTITY='THERMOCOUPLE', XYZ=1.9,0.47,0.47/
&DEVC ID='THCP430', QUANTITY='THERMOCOUPLE', XYZ=1.95,0.47,0.47/
&DEVC ID='THCP431', QUANTITY='THERMOCOUPLE', XYZ=1.0,0.39,0.47/
&DEVC ID='THCP432', QUANTITY='THERMOCOUPLE', XYZ=1.05,0.39,0.47/
&DEVC ID='THCP433', QUANTITY='THERMOCOUPLE', XYZ=1.1,0.39,0.47/
&DEVC ID='THCP434', QUANTITY='THERMOCOUPLE', XYZ=1.15,0.39,0.47/
&DEVC ID='THCP435', QUANTITY='THERMOCOUPLE', XYZ=1.2,0.39,0.47/
&DEVC ID='THCP436', QUANTITY='THERMOCOUPLE', XYZ=1.25,0.39,0.47/
&DEVC ID='THCP437', QUANTITY='THERMOCOUPLE', XYZ=1.3,0.39,0.47/
&DEVC ID='THCP438', QUANTITY='THERMOCOUPLE', XYZ=1.35,0.39,0.47/
&DEVC ID='THCP439', QUANTITY='THERMOCOUPLE', XYZ=1.4,0.39,0.47/
&DEVC ID='THCP440', QUANTITY='THERMOCOUPLE', XYZ=1.45,0.39,0.47/
&DEVC ID='THCP441', QUANTITY='THERMOCOUPLE', XYZ=1.5,0.39,0.47/
&DEVC ID='THCP442', QUANTITY='THERMOCOUPLE', XYZ=1.55,0.39,0.47/
&DEVC ID='THCP443', QUANTITY='THERMOCOUPLE', XYZ=1.6,0.39,0.47/
&DEVC ID='THCP444', QUANTITY='THERMOCOUPLE', XYZ=1.65,0.39,0.47/
&DEVC ID='THCP445', QUANTITY='THERMOCOUPLE', XYZ=1.7,0.39,0.47/
&DEVC ID='THCP446', QUANTITY='THERMOCOUPLE', XYZ=1.75,0.39,0.47/
&DEVC ID='THCP447', QUANTITY='THERMOCOUPLE', XYZ=1.8,0.39,0.47/
&DEVC ID='THCP448', QUANTITY='THERMOCOUPLE', XYZ=1.85,0.39,0.47/
&DEVC ID='THCP449', QUANTITY='THERMOCOUPLE', XYZ=1.9,0.39,0.47/
&DEVC ID='THCP450', QUANTITY='THERMOCOUPLE', XYZ=1.95,0.39,0.47/

```
&DEVC ID='THCP451', QUANTITY='THERMOCOUPLE', XYZ=1.0,0.3,0.47/
&DEVC ID='THCP452', QUANTITY='THERMOCOUPLE', XYZ=1.05,0.3,0.47/
&DEVC ID='THCP453', QUANTITY='THERMOCOUPLE', XYZ=1.1,0.3,0.47/
&DEVC ID='THCP454', QUANTITY='THERMOCOUPLE', XYZ=1.15,0.3,0.47/
&DEVC ID='THCP455', QUANTITY='THERMOCOUPLE', XYZ=1.2,0.3,0.47/
&DEVC ID='THCP456', QUANTITY='THERMOCOUPLE', XYZ=1.25,0.3,0.47/
&DEVC ID='THCP457', QUANTITY='THERMOCOUPLE', XYZ=1.3,0.3,0.47/
&DEVC ID='THCP458', QUANTITY='THERMOCOUPLE', XYZ=1.35,0.3,0.47/
&DEVC ID='THCP459', QUANTITY='THERMOCOUPLE', XYZ=1.4,0.3,0.47/
&DEVC ID='THCP460', QUANTITY='THERMOCOUPLE', XYZ=1.45,0.3,0.47/
&DEVC ID='THCP461', QUANTITY='THERMOCOUPLE', XYZ=1.5,0.3,0.47/
&DEVC ID='THCP462', QUANTITY='THERMOCOUPLE', XYZ=1.55,0.3,0.47/
&DEVC ID='THCP463', QUANTITY='THERMOCOUPLE', XYZ=1.6,0.3,0.47/
&DEVC ID='THCP464', QUANTITY='THERMOCOUPLE', XYZ=1.65,0.3,0.47/
&DEVC ID='THCP465', QUANTITY='THERMOCOUPLE', XYZ=1.7,0.3,0.47/
&DEVC ID='THCP466', QUANTITY='THERMOCOUPLE', XYZ=1.75,0.3,0.47/
&DEVC ID='THCP467', QUANTITY='THERMOCOUPLE', XYZ=1.8,0.3,0.47/
&DEVC ID='THCP468', QUANTITY='THERMOCOUPLE', XYZ=1.85,0.3,0.47/
&DEVC ID='THCP469', QUANTITY='THERMOCOUPLE', XYZ=1.9,0.3,0.47/
&DEVC ID='THCP470', QUANTITY='THERMOCOUPLE', XYZ=1.95,0.3,0.47/
&DEVC ID='THCP471', QUANTITY='THERMOCOUPLE', XYZ=1.0,0.21,0.47/
&DEVC ID='THCP472', QUANTITY='THERMOCOUPLE', XYZ=1.05,0.21,0.47/
&DEVC ID='THCP473', QUANTITY='THERMOCOUPLE', XYZ=1.1,0.21,0.47/
&DEVC ID='THCP474', QUANTITY='THERMOCOUPLE', XYZ=1.15,0.21,0.47/
&DEVC ID='THCP475', QUANTITY='THERMOCOUPLE', XYZ=1.2,0.21,0.47/
&DEVC ID='THCP476', QUANTITY='THERMOCOUPLE', XYZ=1.25,0.21,0.47/
&DEVC ID='THCP477', QUANTITY='THERMOCOUPLE', XYZ=1.3,0.21,0.47/
&DEVC ID='THCP478', QUANTITY='THERMOCOUPLE', XYZ=1.35,0.21,0.47/
&DEVC ID='THCP479', QUANTITY='THERMOCOUPLE', XYZ=1.4,0.21,0.47/
&DEVC ID='THCP480', QUANTITY='THERMOCOUPLE', XYZ=1.45,0.21,0.47/
&DEVC ID='THCP481', QUANTITY='THERMOCOUPLE', XYZ=1.5,0.21,0.47/
&DEVC ID='THCP482', QUANTITY='THERMOCOUPLE', XYZ=1.55,0.21,0.47/
&DEVC ID='THCP483', QUANTITY='THERMOCOUPLE', XYZ=1.6,0.21,0.47/
&DEVC ID='THCP484', QUANTITY='THERMOCOUPLE', XYZ=1.65,0.21,0.47/
&DEVC ID='THCP485', QUANTITY='THERMOCOUPLE', XYZ=1.7,0.21,0.47/
&DEVC ID='THCP486', QUANTITY='THERMOCOUPLE', XYZ=1.75,0.21,0.47/
&DEVC ID='THCP487', QUANTITY='THERMOCOUPLE', XYZ=1.8,0.21,0.47/
&DEVC ID='THCP488', QUANTITY='THERMOCOUPLE', XYZ=1.85,0.21,0.47/
&DEVC ID='THCP489', QUANTITY='THERMOCOUPLE', XYZ=1.9,0.21,0.47/
&DEVC ID='THCP490', QUANTITY='THERMOCOUPLE', XYZ=1.95,0.21,0.47/
&DEVC ID='THCP491', QUANTITY='THERMOCOUPLE', XYZ=1.0,0.12,0.47/
&DEVC ID='THCP492', QUANTITY='THERMOCOUPLE', XYZ=1.05,0.12,0.47/
&DEVC ID='THCP493', QUANTITY='THERMOCOUPLE', XYZ=1.1,0.12,0.47/
&DEVC ID='THCP494', QUANTITY='THERMOCOUPLE', XYZ=1.15,0.12,0.47/
```

```
&DEVC ID='THCP495', QUANTITY='THERMOCOUPLE', XYZ=1.2,0.12,0.47/
&DEVC ID='THCP496', QUANTITY='THERMOCOUPLE', XYZ=1.25,0.12,0.47/
&DEVC ID='THCP497', QUANTITY='THERMOCOUPLE', XYZ=1.3,0.12,0.47/
&DEVC ID='THCP498', QUANTITY='THERMOCOUPLE', XYZ=1.35,0.12,0.47/
&DEVC ID='THCP499', QUANTITY='THERMOCOUPLE', XYZ=1.4,0.12,0.47/
&DEVC ID='THCP500', QUANTITY='THERMOCOUPLE', XYZ=1.45,0.12,0.47/
&DEVC ID='THCP501', QUANTITY='THERMOCOUPLE', XYZ=1.5,0.12,0.47/
&DEVC ID='THCP502', QUANTITY='THERMOCOUPLE', XYZ=1.55,0.12,0.47/
&DEVC ID='THCP503', QUANTITY='THERMOCOUPLE', XYZ=1.6,0.12,0.47/
&DEVC ID='THCP504', QUANTITY='THERMOCOUPLE', XYZ=1.65,0.12,0.47/
&DEVC ID='THCP505', QUANTITY='THERMOCOUPLE', XYZ=1.7,0.12,0.47/
&DEVC ID='THCP506', QUANTITY='THERMOCOUPLE', XYZ=1.75,0.12,0.47/
&DEVC ID='THCP507', QUANTITY='THERMOCOUPLE', XYZ=1.8,0.12,0.47/
&DEVC ID='THCP508', QUANTITY='THERMOCOUPLE', XYZ=1.85,0.12,0.47/
&DEVC ID='THCP509', QUANTITY='THERMOCOUPLE', XYZ=1.9,0.12,0.47/
&DEVC ID='THCP510', QUANTITY='THERMOCOUPLE', XYZ=1.95,0.12,0.47/
&DEVC ID='THCP511', QUANTITY='THERMOCOUPLE', XYZ=1.0,0.03,0.47/
&DEVC ID='THCP512', QUANTITY='THERMOCOUPLE', XYZ=1.05,0.03,0.47/
&DEVC ID='THCP513', QUANTITY='THERMOCOUPLE', XYZ=1.1,0.03,0.47/
&DEVC ID='THCP514', QUANTITY='THERMOCOUPLE', XYZ=1.15,0.03,0.47/
&DEVC ID='THCP515', QUANTITY='THERMOCOUPLE', XYZ=1.2,0.03,0.47/
&DEVC ID='THCP516', QUANTITY='THERMOCOUPLE', XYZ=1.25,0.03,0.47/
&DEVC ID='THCP517', QUANTITY='THERMOCOUPLE', XYZ=1.3,0.03,0.47/
&DEVC ID='THCP518', QUANTITY='THERMOCOUPLE', XYZ=1.35,0.03,0.47/
&DEVC ID='THCP519', QUANTITY='THERMOCOUPLE', XYZ=1.4,0.03,0.47/
&DEVC ID='THCP520', QUANTITY='THERMOCOUPLE', XYZ=1.45,0.03,0.47/
&DEVC ID='THCP521', QUANTITY='THERMOCOUPLE', XYZ=1.5,0.03,0.47/
&DEVC ID='THCP522', QUANTITY='THERMOCOUPLE', XYZ=1.55,0.03,0.47/
&DEVC ID='THCP523', QUANTITY='THERMOCOUPLE', XYZ=1.6,0.03,0.47/
&DEVC ID='THCP524', QUANTITY='THERMOCOUPLE', XYZ=1.65,0.03,0.47/
&DEVC ID='THCP525', QUANTITY='THERMOCOUPLE', XYZ=1.7,0.03,0.47/
&DEVC ID='THCP526', QUANTITY='THERMOCOUPLE', XYZ=1.75,0.03,0.47/
&DEVC ID='THCP527', QUANTITY='THERMOCOUPLE', XYZ=1.8,0.03,0.47/
&DEVC ID='THCP528', QUANTITY='THERMOCOUPLE', XYZ=1.85,0.03,0.47/
&DEVC ID='THCP529', QUANTITY='THERMOCOUPLE', XYZ=1.9,0.03,0.47/
&DEVC ID='THCP531', QUANTITY='THERMOCOUPLE', XYZ=1.0,0.03,0.0/
&DEVC ID='THCP532', QUANTITY='THERMOCOUPLE', XYZ=1.05,0.03,0.0/
&DEVC ID='THCP533', QUANTITY='THERMOCOUPLE', XYZ=1.1,0.03,0.0/
&DEVC ID='THCP534', QUANTITY='THERMOCOUPLE', XYZ=1.15,0.03,0.0/
&DEVC ID='THCP535', QUANTITY='THERMOCOUPLE', XYZ=1.2,0.03,0.0/
&DEVC ID='THCP536', QUANTITY='THERMOCOUPLE', XYZ=1.25,0.03,0.0/
&DEVC ID='THCP537', QUANTITY='THERMOCOUPLE', XYZ=1.3,0.03,0.0/
&DEVC ID='THCP538', QUANTITY='THERMOCOUPLE', XYZ=1.35,0.03,0.0/
&DEVC ID='THCP539', QUANTITY='THERMOCOUPLE', XYZ=1.4,0.03,0.0/
```

```
&DEVC ID='THCP540', QUANTITY='THERMOCOUPLE', XYZ=1.45,0.03,0.0/
&DEVC ID='THCP541', QUANTITY='THERMOCOUPLE', XYZ=1.5,0.03,0.0/
&DEVC ID='THCP542', QUANTITY='THERMOCOUPLE', XYZ=1.55,0.03,0.0/
&DEVC ID='THCP543', QUANTITY='THERMOCOUPLE', XYZ=1.6,0.03,0.0/
&DEVC ID='THCP544', QUANTITY='THERMOCOUPLE', XYZ=1.65,0.03,0.0/
&DEVC ID='THCP545', QUANTITY='THERMOCOUPLE', XYZ=1.7,0.03,0.0/
&DEVC ID='THCP546', QUANTITY='THERMOCOUPLE', XYZ=1.75,0.03,0.0/
&DEVC ID='THCP547', QUANTITY='THERMOCOUPLE', XYZ=1.8,0.03,0.0/
&DEVC ID='THCP548', QUANTITY='THERMOCOUPLE', XYZ=1.85,0.03,0.0/
&DEVC ID='THCP549', QUANTITY='THERMOCOUPLE', XYZ=1.9,0.03,0.0/
&DEVC ID='THCP550', QUANTITY='THERMOCOUPLE', XYZ=1.95,0.03,0.0/
&DEVC ID='THCP551', QUANTITY='THERMOCOUPLE', XYZ=1.0,0.03,0.05/
&DEVC ID='THCP552', QUANTITY='THERMOCOUPLE', XYZ=1.05,0.03,0.05/
&DEVC ID='THCP553', QUANTITY='THERMOCOUPLE', XYZ=1.1,0.03,0.05/
&DEVC ID='THCP554', QUANTITY='THERMOCOUPLE', XYZ=1.15,0.03,0.05/
&DEVC ID='THCP555', QUANTITY='THERMOCOUPLE', XYZ=1.2,0.03,0.05/
&DEVC ID='THCP556', QUANTITY='THERMOCOUPLE', XYZ=1.25,0.03,0.05/
&DEVC ID='THCP557', QUANTITY='THERMOCOUPLE', XYZ=1.3,0.03,0.05/
&DEVC ID='THCP558', QUANTITY='THERMOCOUPLE', XYZ=1.35,0.03,0.05/
&DEVC ID='THCP559', QUANTITY='THERMOCOUPLE', XYZ=1.4,0.03,0.05/
&DEVC ID='THCP560', QUANTITY='THERMOCOUPLE', XYZ=1.45,0.03,0.05/
&DEVC ID='THCP561', QUANTITY='THERMOCOUPLE', XYZ=1.5,0.03,0.05/
&DEVC ID='THCP562', QUANTITY='THERMOCOUPLE', XYZ=1.55,0.03,0.05/
&DEVC ID='THCP563', QUANTITY='THERMOCOUPLE', XYZ=1.6,0.03,0.05/
&DEVC ID='THCP564', QUANTITY='THERMOCOUPLE', XYZ=1.65,0.03,0.05/
&DEVC ID='THCP565', QUANTITY='THERMOCOUPLE', XYZ=1.7,0.03,0.05/
&DEVC ID='THCP566', QUANTITY='THERMOCOUPLE', XYZ=1.75,0.03,0.05/
&DEVC ID='THCP567', QUANTITY='THERMOCOUPLE', XYZ=1.8,0.03,0.05/
&DEVC ID='THCP568', QUANTITY='THERMOCOUPLE', XYZ=1.85,0.03,0.05/
&DEVC ID='THCP569', QUANTITY='THERMOCOUPLE', XYZ=1.9,0.03,0.05/
&DEVC ID='THCP570', QUANTITY='THERMOCOUPLE', XYZ=1.95,0.03,0.05/
&DEVC ID='THCP571', QUANTITY='THERMOCOUPLE', XYZ=1.0,0.03,0.1/
&DEVC ID='THCP572', QUANTITY='THERMOCOUPLE', XYZ=1.05,0.03,0.1/
&DEVC ID='THCP573', QUANTITY='THERMOCOUPLE', XYZ=1.1,0.03,0.1/
&DEVC ID='THCP574', QUANTITY='THERMOCOUPLE', XYZ=1.15,0.03,0.1/
&DEVC ID='THCP575', QUANTITY='THERMOCOUPLE', XYZ=1.2,0.03,0.1/
&DEVC ID='THCP576', QUANTITY='THERMOCOUPLE', XYZ=1.25,0.03,0.1/
&DEVC ID='THCP577', QUANTITY='THERMOCOUPLE', XYZ=1.3,0.03,0.1/
&DEVC ID='THCP578', QUANTITY='THERMOCOUPLE', XYZ=1.35,0.03,0.1/
&DEVC ID='THCP579', QUANTITY='THERMOCOUPLE', XYZ=1.4,0.03,0.1/
&DEVC ID='THCP580', QUANTITY='THERMOCOUPLE', XYZ=1.45,0.03,0.1/
&DEVC ID='THCP581', QUANTITY='THERMOCOUPLE', XYZ=1.5,0.03,0.1/
&DEVC ID='THCP582', QUANTITY='THERMOCOUPLE', XYZ=1.55,0.03,0.1/
&DEVC ID='THCP583', QUANTITY='THERMOCOUPLE', XYZ=1.6,0.03,0.1/
```

```
&DEVC ID='THCP584', QUANTITY='THERMOCOUPLE', XYZ=1.65,0.03,0.1/
&DEVC ID='THCP585', QUANTITY='THERMOCOUPLE', XYZ=1.7,0.03,0.1/
&DEVC ID='THCP586', QUANTITY='THERMOCOUPLE', XYZ=1.75,0.03,0.1/
&DEVC ID='THCP587', QUANTITY='THERMOCOUPLE', XYZ=1.8,0.03,0.1/
&DEVC ID='THCP588', QUANTITY='THERMOCOUPLE', XYZ=1.85,0.03,0.1/
&DEVC ID='THCP589', QUANTITY='THERMOCOUPLE', XYZ=1.9,0.03,0.1/
&DEVC ID='THCP590', QUANTITY='THERMOCOUPLE', XYZ=1.95,0.03,0.1/
&DEVC ID='THCP591', QUANTITY='THERMOCOUPLE', XYZ=1.0,0.03,0.15/
&DEVC ID='THCP592', QUANTITY='THERMOCOUPLE', XYZ=1.05,0.03,0.15/
&DEVC ID='THCP593', QUANTITY='THERMOCOUPLE', XYZ=1.1,0.03,0.15/
&DEVC ID='THCP594', QUANTITY='THERMOCOUPLE', XYZ=1.15,0.03,0.15/
&DEVC ID='THCP595', QUANTITY='THERMOCOUPLE', XYZ=1.2,0.03,0.15/
&DEVC ID='THCP596', QUANTITY='THERMOCOUPLE', XYZ=1.25,0.03,0.15/
&DEVC ID='THCP597', QUANTITY='THERMOCOUPLE', XYZ=1.3,0.03,0.15/
&DEVC ID='THCP598', QUANTITY='THERMOCOUPLE', XYZ=1.35,0.03,0.15/
&DEVC ID='THCP599', QUANTITY='THERMOCOUPLE', XYZ=1.4,0.03,0.15/
&DEVC ID='THCP600', QUANTITY='THERMOCOUPLE', XYZ=1.45,0.03,0.15/
&DEVC ID='THCP601', QUANTITY='THERMOCOUPLE', XYZ=1.5,0.03,0.15/
&DEVC ID='THCP602', QUANTITY='THERMOCOUPLE', XYZ=1.55,0.03,0.15/
&DEVC ID='THCP603', QUANTITY='THERMOCOUPLE', XYZ=1.6,0.03,0.15/
&DEVC ID='THCP604', QUANTITY='THERMOCOUPLE', XYZ=1.65,0.03,0.15/
&DEVC ID='THCP605', QUANTITY='THERMOCOUPLE', XYZ=1.7,0.03,0.15/
&DEVC ID='THCP606', QUANTITY='THERMOCOUPLE', XYZ=1.75,0.03,0.15/
&DEVC ID='THCP607', QUANTITY='THERMOCOUPLE', XYZ=1.8,0.03,0.15/
&DEVC ID='THCP608', QUANTITY='THERMOCOUPLE', XYZ=1.85,0.03,0.15/
&DEVC ID='THCP609', QUANTITY='THERMOCOUPLE', XYZ=1.9,0.03,0.15/
&DEVC ID='THCP610', QUANTITY='THERMOCOUPLE', XYZ=1.95,0.03,0.15/
&DEVC ID='THCP611', QUANTITY='THERMOCOUPLE', XYZ=1.0,0.03,0.2/
&DEVC ID='THCP612', QUANTITY='THERMOCOUPLE', XYZ=1.05,0.03,0.2/
&DEVC ID='THCP613', QUANTITY='THERMOCOUPLE', XYZ=1.1,0.03,0.2/
&DEVC ID='THCP614', QUANTITY='THERMOCOUPLE', XYZ=1.15,0.03,0.2/
&DEVC ID='THCP615', QUANTITY='THERMOCOUPLE', XYZ=1.2,0.03,0.2/
&DEVC ID='THCP616', QUANTITY='THERMOCOUPLE', XYZ=1.25,0.03,0.2/
&DEVC ID='THCP617', QUANTITY='THERMOCOUPLE', XYZ=1.3,0.03,0.2/
&DEVC ID='THCP618', QUANTITY='THERMOCOUPLE', XYZ=1.35,0.03,0.2/
&DEVC ID='THCP619', QUANTITY='THERMOCOUPLE', XYZ=1.4,0.03,0.2/
&DEVC ID='THCP620', QUANTITY='THERMOCOUPLE', XYZ=1.45,0.03,0.2/
&DEVC ID='THCP621', QUANTITY='THERMOCOUPLE', XYZ=1.5,0.03,0.2/
&DEVC ID='THCP622', QUANTITY='THERMOCOUPLE', XYZ=1.55,0.03,0.2/
&DEVC ID='THCP623', QUANTITY='THERMOCOUPLE', XYZ=1.6,0.03,0.2/
&DEVC ID='THCP624', QUANTITY='THERMOCOUPLE', XYZ=1.65,0.03,0.2/
&DEVC ID='THCP625', QUANTITY='THERMOCOUPLE', XYZ=1.7,0.03,0.2/
&DEVC ID='THCP626', QUANTITY='THERMOCOUPLE', XYZ=1.75,0.03,0.2/
&DEVC ID='THCP627', QUANTITY='THERMOCOUPLE', XYZ=1.8,0.03,0.2/
```

```
&DEVC ID='THCP628', QUANTITY='THERMOCOUPLE', XYZ=1.85,0.03,0.2/
&DEVC ID='THCP629', QUANTITY='THERMOCOUPLE', XYZ=1.9,0.03,0.2/
&DEVC ID='THCP630', QUANTITY='THERMOCOUPLE', XYZ=1.95,0.03,0.2/
&DEVC ID='THCP631', QUANTITY='THERMOCOUPLE', XYZ=1.0,0.03,0.25/
&DEVC ID='THCP632', QUANTITY='THERMOCOUPLE', XYZ=1.05,0.03,0.25/
&DEVC ID='THCP633', QUANTITY='THERMOCOUPLE', XYZ=1.1,0.03,0.25/
&DEVC ID='THCP634', QUANTITY='THERMOCOUPLE', XYZ=1.15,0.03,0.25/
&DEVC ID='THCP635', QUANTITY='THERMOCOUPLE', XYZ=1.2,0.03,0.25/
&DEVC ID='THCP636', QUANTITY='THERMOCOUPLE', XYZ=1.25,0.03,0.25/
&DEVC ID='THCP637', QUANTITY='THERMOCOUPLE', XYZ=1.3,0.03,0.25/
&DEVC ID='THCP638', QUANTITY='THERMOCOUPLE', XYZ=1.35,0.03,0.25/
&DEVC ID='THCP639', QUANTITY='THERMOCOUPLE', XYZ=1.4,0.03,0.25/
&DEVC ID='THCP640', QUANTITY='THERMOCOUPLE', XYZ=1.45,0.03,0.25/
&DEVC ID='THCP641', QUANTITY='THERMOCOUPLE', XYZ=1.5,0.03,0.25/
&DEVC ID='THCP642', QUANTITY='THERMOCOUPLE', XYZ=1.55,0.03,0.25/
&DEVC ID='THCP643', QUANTITY='THERMOCOUPLE', XYZ=1.6,0.03,0.25/
&DEVC ID='THCP644', QUANTITY='THERMOCOUPLE', XYZ=1.65,0.03,0.25/
&DEVC ID='THCP645', QUANTITY='THERMOCOUPLE', XYZ=1.7,0.03,0.25/
&DEVC ID='THCP646', QUANTITY='THERMOCOUPLE', XYZ=1.75,0.03,0.25/
&DEVC ID='THCP647', QUANTITY='THERMOCOUPLE', XYZ=1.8,0.03,0.25/
&DEVC ID='THCP648', QUANTITY='THERMOCOUPLE', XYZ=1.85,0.03,0.25/
&DEVC ID='THCP649', QUANTITY='THERMOCOUPLE', XYZ=1.9,0.03,0.25/
&DEVC ID='THCP650', QUANTITY='THERMOCOUPLE', XYZ=1.95,0.03,0.25/
&DEVC ID='THCP651', QUANTITY='THERMOCOUPLE', XYZ=1.0,0.03,0.3/
&DEVC ID='THCP652', QUANTITY='THERMOCOUPLE', XYZ=1.05,0.03,0.3/
&DEVC ID='THCP653', QUANTITY='THERMOCOUPLE', XYZ=1.1,0.03,0.3/
&DEVC ID='THCP654', QUANTITY='THERMOCOUPLE', XYZ=1.15,0.03,0.3/
&DEVC ID='THCP655', QUANTITY='THERMOCOUPLE', XYZ=1.2,0.03,0.3/
&DEVC ID='THCP656', QUANTITY='THERMOCOUPLE', XYZ=1.25,0.03,0.3/
&DEVC ID='THCP657', QUANTITY='THERMOCOUPLE', XYZ=1.3,0.03,0.3/
&DEVC ID='THCP658', QUANTITY='THERMOCOUPLE', XYZ=1.35,0.03,0.3/
&DEVC ID='THCP659', QUANTITY='THERMOCOUPLE', XYZ=1.4,0.03,0.3/
&DEVC ID='THCP660', QUANTITY='THERMOCOUPLE', XYZ=1.45,0.03,0.3/
&DEVC ID='THCP661', QUANTITY='THERMOCOUPLE', XYZ=1.5,0.03,0.3/
&DEVC ID='THCP662', QUANTITY='THERMOCOUPLE', XYZ=1.55,0.03,0.3/
&DEVC ID='THCP663', QUANTITY='THERMOCOUPLE', XYZ=1.6,0.03,0.3/
&DEVC ID='THCP664', QUANTITY='THERMOCOUPLE', XYZ=1.65,0.03,0.3/
&DEVC ID='THCP665', QUANTITY='THERMOCOUPLE', XYZ=1.7,0.03,0.3/
&DEVC ID='THCP666', QUANTITY='THERMOCOUPLE', XYZ=1.75,0.03,0.3/
&DEVC ID='THCP667', QUANTITY='THERMOCOUPLE', XYZ=1.8,0.03,0.3/
&DEVC ID='THCP668', QUANTITY='THERMOCOUPLE', XYZ=1.85,0.03,0.3/
&DEVC ID='THCP669', QUANTITY='THERMOCOUPLE', XYZ=1.9,0.03,0.3/
&DEVC ID='THCP670', QUANTITY='THERMOCOUPLE', XYZ=1.95,0.03,0.3/
&DEVC ID='THCP671', QUANTITY='THERMOCOUPLE', XYZ=1.0,0.03,0.35/
```

```
&DEVC ID='THCP672', QUANTITY='THERMOCOUPLE', XYZ=1.05,0.03,0.35/
&DEVC ID='THCP673', QUANTITY='THERMOCOUPLE', XYZ=1.1,0.03,0.35/
&DEVC ID='THCP674', QUANTITY='THERMOCOUPLE', XYZ=1.15,0.03,0.35/
&DEVC ID='THCP675', QUANTITY='THERMOCOUPLE', XYZ=1.2,0.03,0.35/
&DEVC ID='THCP676', QUANTITY='THERMOCOUPLE', XYZ=1.25,0.03,0.35/
&DEVC ID='THCP677', QUANTITY='THERMOCOUPLE', XYZ=1.3,0.03,0.35/
&DEVC ID='THCP678', QUANTITY='THERMOCOUPLE', XYZ=1.35,0.03,0.35/
&DEVC ID='THCP679', QUANTITY='THERMOCOUPLE', XYZ=1.4,0.03,0.35/
&DEVC ID='THCP680', QUANTITY='THERMOCOUPLE', XYZ=1.45,0.03,0.35/
&DEVC ID='THCP681', QUANTITY='THERMOCOUPLE', XYZ=1.5,0.03,0.35/
&DEVC ID='THCP682', QUANTITY='THERMOCOUPLE', XYZ=1.55,0.03,0.35/
&DEVC ID='THCP683', QUANTITY='THERMOCOUPLE', XYZ=1.6,0.03,0.35/
&DEVC ID='THCP684', QUANTITY='THERMOCOUPLE', XYZ=1.65,0.03,0.35/
&DEVC ID='THCP685', QUANTITY='THERMOCOUPLE', XYZ=1.7,0.03,0.35/
&DEVC ID='THCP686', QUANTITY='THERMOCOUPLE', XYZ=1.75,0.03,0.35/
&DEVC ID='THCP687', QUANTITY='THERMOCOUPLE', XYZ=1.8,0.03,0.35/
&DEVC ID='THCP688', QUANTITY='THERMOCOUPLE', XYZ=1.85,0.03,0.35/
&DEVC ID='THCP689', QUANTITY='THERMOCOUPLE', XYZ=1.9,0.03,0.35/
&DEVC ID='THCP690', QUANTITY='THERMOCOUPLE', XYZ=1.95,0.03,0.35/
&DEVC ID='THCP691', QUANTITY='THERMOCOUPLE', XYZ=1.0,0.03,0.4/
&DEVC ID='THCP692', QUANTITY='THERMOCOUPLE', XYZ=1.05,0.03,0.4/
&DEVC ID='THCP693', QUANTITY='THERMOCOUPLE', XYZ=1.1,0.03,0.4/
&DEVC ID='THCP694', QUANTITY='THERMOCOUPLE', XYZ=1.15,0.03,0.4/
&DEVC ID='THCP695', QUANTITY='THERMOCOUPLE', XYZ=1.2,0.03,0.4/
&DEVC ID='THCP696', QUANTITY='THERMOCOUPLE', XYZ=1.25,0.03,0.4/
&DEVC ID='THCP697', QUANTITY='THERMOCOUPLE', XYZ=1.3,0.03,0.4/
&DEVC ID='THCP698', QUANTITY='THERMOCOUPLE', XYZ=1.35,0.03,0.4/
&DEVC ID='THCP699', QUANTITY='THERMOCOUPLE', XYZ=1.4,0.03,0.4/
&DEVC ID='THCP700', QUANTITY='THERMOCOUPLE', XYZ=1.45,0.03,0.4/
&DEVC ID='THCP701', QUANTITY='THERMOCOUPLE', XYZ=1.5,0.03,0.4/
&DEVC ID='THCP702', QUANTITY='THERMOCOUPLE', XYZ=1.55,0.03,0.4/
&DEVC ID='THCP703', QUANTITY='THERMOCOUPLE', XYZ=1.6,0.03,0.4/
&DEVC ID='THCP704', QUANTITY='THERMOCOUPLE', XYZ=1.65,0.03,0.4/
&DEVC ID='THCP705', QUANTITY='THERMOCOUPLE', XYZ=1.7,0.03,0.4/
&DEVC ID='THCP706', QUANTITY='THERMOCOUPLE', XYZ=1.75,0.03,0.4/
&DEVC ID='THCP707', QUANTITY='THERMOCOUPLE', XYZ=1.8,0.03,0.4/
&DEVC ID='THCP708', QUANTITY='THERMOCOUPLE', XYZ=1.85,0.03,0.4/
&DEVC ID='THCP709', QUANTITY='THERMOCOUPLE', XYZ=1.9,0.03,0.4/
&DEVC ID='THCP710', QUANTITY='THERMOCOUPLE', XYZ=1.95,0.03,0.4/

&MATL ID='STEEL',
      FYI='Drysdale, Intro to Fire Dynamics - ATF NIST Multi-Floor Validation',
      SPECIFIC_HEAT=0.46,
      CONDUCTIVITY=45.8,
```

```
          DENSITY=7850.0,
          EMISSIVITY=0.95/
&MATL ID='GYPSUM PLASTER',
          FYI='Quintiere, Fire Behavior - NIST NRC Validation',
          SPECIFIC_HEAT=0.84,
          CONDUCTIVITY=0.48,
          DENSITY=1440.0/
&MATL ID='YELLOW PINE',
          FYI='Quintiere, Fire Behavior - NIST NRC Validation',
          SPECIFIC_HEAT=2.85,
          CONDUCTIVITY=0.14,
          DENSITY=640.0,
          HEAT_OF_COMBUSTION=1.64E4,
          N_REACTIONS=1,
          MATL_ID(1,1)='ash',
          NU_MATL(1,1)=0.1,
          SPEC_ID(1,1)='REAC_FUEL',
          NU_SPEC(1,1)=0.9,
          REFERENCE_TEMPERATURE=332.0/
&MATL ID='ash',
          SPECIFIC_HEAT=1.0,
          CONDUCTIVITY=0.2,
          DENSITY=500.0,
          EMISSIVITY=1.0/

&SURF ID='铁板',
          COLOR='GRAY 20',
          DEFAULT=.TRUE.,
          BACKING='VOID',
          MATL_ID(1,1)='STEEL',
          MATL_MASS_FRACTION(1,1)=1.0,
          THICKNESS(1)=5.0E-3/
&SURF ID='fire',
          COLOR='RED',
          HRRPUA=800.0,
          RAMP_Q='fire_RAMP_Q',
          TMP_FRONT=300.0/
&RAMP ID='fire_RAMP_Q', T=0.0, F=0.0/
&RAMP ID='fire_RAMP_Q', T=200.0, F=1.0/
&RAMP ID='fire_RAMP_Q', T=500.0, F=1.0/
&RAMP ID='fire_RAMP_Q', T=700.0, F=0.0/
&SURF ID='石膏板',
          COLOR='GRAY 80',
          BACKING='VOID',
```

```
	MATL_ID(1,1)='GYPSUM PLASTER',
	MATL_MASS_FRACTION(1,1)=1.0,
	THICKNESS(1)=0.015/
&SURF ID='石膏-木板',
	RGB=146,202,166,
	TEXTURE_MAP='psm_spruce.jpg',
	TEXTURE_WIDTH=0.67056,
	TEXTURE_HEIGHT=2.4384,
	BACKING='VOID',
	MATL_ID(1,1)='YELLOW PINE',
	MATL_MASS_FRACTION(1,1)=1.0,
	THICKNESS(1)=0.01/

&OBST ID='火源', XB=1.92,2.0,0.04,0.06,0.04,0.12, SURF_ID6='INERT','INERT','fire','INERT','INERT','INERT'/
&OBST ID='Obstruction', XB=-0.5,4.5,-0.02,0.52,-0.02,0.0, SURF_IDS='石膏板','石膏板','铁板'/
&OBST ID='Obstruction', XB=-0.5,4.5,-0.02,0.52,0.5,0.52, SURF_IDS='铁板','石膏板','石膏板'/
&OBST ID='Obstruction', XB=-0.5,4.5,-0.02,0.0,0.0,0.5, COLOR='INVISIBLE', SURF_ID6='石膏板','石膏板','铁板','石膏板','石膏板','石膏板'/
&OBST ID='Obstruction', XB=-0.5,4.5,0.5,0.52,0.0,0.5, COLOR='INVISIBLE', SURF_ID6='石膏板','石膏板','石膏板','铁板','石膏板','石膏板'/
&OBST ID='Obstruction', XB=1.0,1.25,0.0,0.5,0.48,0.5, SURF_IDS='石膏板','石膏板','石膏-木板'/
&OBST ID='Obstruction', XB=1.25,1.5,0.0,0.5,0.48,0.5, SURF_IDS='石膏板','石膏板','石膏-木板'/
&OBST ID='Obstruction', XB=1.5,1.75,0.0,0.5,0.48,0.5, SURF_IDS='石膏板','石膏板','石膏-木板'/
&OBST ID='Obstruction', XB=1.75,2.0,0.0,0.5,0.48,0.5, SURF_IDS='石膏板','石膏板','石膏-木板'/
&OBST ID='Obstruction', XB=1.0,1.25,0.0,0.02,0.0,0.48, SURF_ID6='石膏板','石膏板','石膏板','石膏-木板','石膏板','石膏板'/
&OBST ID='Obstruction', XB=1.0,1.25,0.48,0.5,0.0,0.48, SURF_ID6='石膏板','石膏板','石膏-木板','石膏板','石膏板','石膏板'/
&OBST ID='Obstruction', XB=1.25,1.5,0.0,0.02,0.0,0.48, SURF_ID6='石膏板','石膏板','石膏板','石膏-木板','石膏板','石膏板'/
&OBST ID='Obstruction', XB=1.25,1.5,0.48,0.5,0.0,0.48, SURF_ID6='石膏板','石膏板','石膏-木板','石膏板','石膏板','石膏板'/
&OBST ID='Obstruction', XB=1.5,1.75,0.0,0.02,0.0,0.48, SURF_ID6='石膏板','石膏板','石膏板','石膏-木板','石膏板','石膏板'/
&OBST ID='Obstruction', XB=1.5,1.75,0.48,0.5,0.0,0.48, SURF_ID6='石膏板','石膏板','石膏-木板','石膏板','石膏板','石膏板'/
&OBST ID='Obstruction', XB=1.75,2.0,0.0,0.02,0.0,0.48, SURF_ID6='石膏板','石膏板','石膏板','石膏-木板','石膏板','石膏板'/
&OBST ID='Obstruction', XB=1.75,2.0,0.48,0.5,0.0,0.48, SURF_ID6='石膏板','石膏板','石膏-木
```

```
板','石膏板','石膏板','石膏板'/

&VENT ID='Vent03', SURF_ID='OPEN', XB=-0.5,-0.5,0.0,0.5,0.0,0.5, COLOR='INVISIBLE'/
&VENT ID='Vent04', SURF_ID='OPEN', XB=4.5,4.5,0.0,0.5,0.0,0.5/

&BNDF QUANTITY='GAS TEMPERATURE'/
&BNDF QUANTITY='NORMAL VELOCITY'/

&ISOF QUANTITY='PRESSURE', VALUE=0.1,1.0,1.1,1.2,1.4,1.6/

&SLCF QUANTITY='TEMPERATURE', PBY=0.02/
&SLCF QUANTITY='TEMPERATURE', PBY=0.48/
&SLCF QUANTITY='TEMPERATURE', PBZ=0.48/
&SLCF QUANTITY='PRESSURE', VECTOR=.TRUE., XB=0.5,2.5,0.0,0.5,0.0,0.5, FYI='Pressure'/
&SLCF QUANTITY='VELOCITY', VECTOR=.TRUE., XB=0.5,2.5,0.0,0.5,0.0,0.5, FYI='Velocity'/


&TAIL /
```
